# Supplementary material for: Rates of Mitochondrial Metabolism of Glucose, Amino Acids, and Fatty Acids by the HEI-OC1 Inner Ear Cell Line
Source: Biology (Basel). 2025 Aug 24;14(9):1118. doi: 10.3390/biology14091118 (PMC12467209; doi:10.3390/biology14091118)
Supplement: Supplementary file 1 [file biology-14-01118-s001.zip › Suppl.S2 Statistical Analysis/Statistical Analysis Results(Fig.10 ).pdf]

AVG AUC (X–Y)" refers to the average oxygen consumption rate calculated from timepoints X to Y during the plateau phase after substrate or inhibitor addition.

A

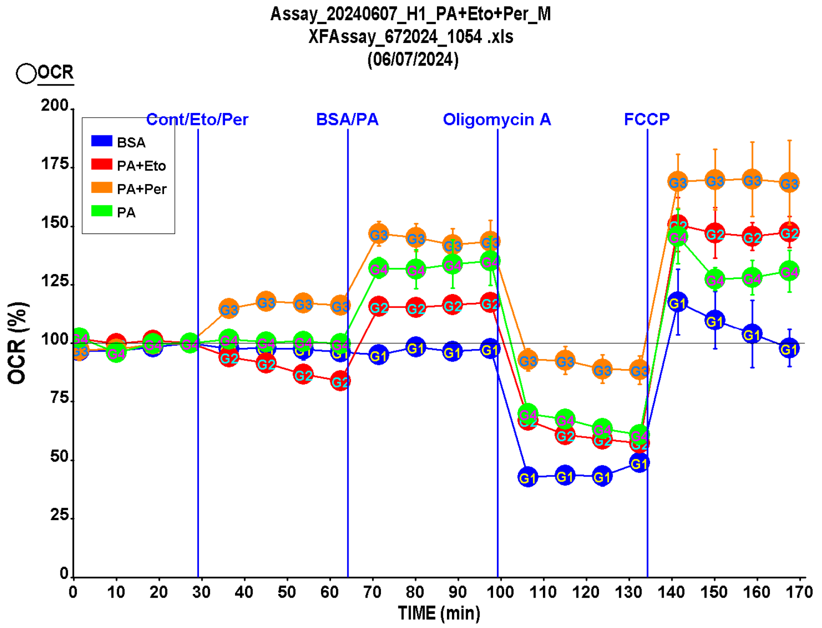

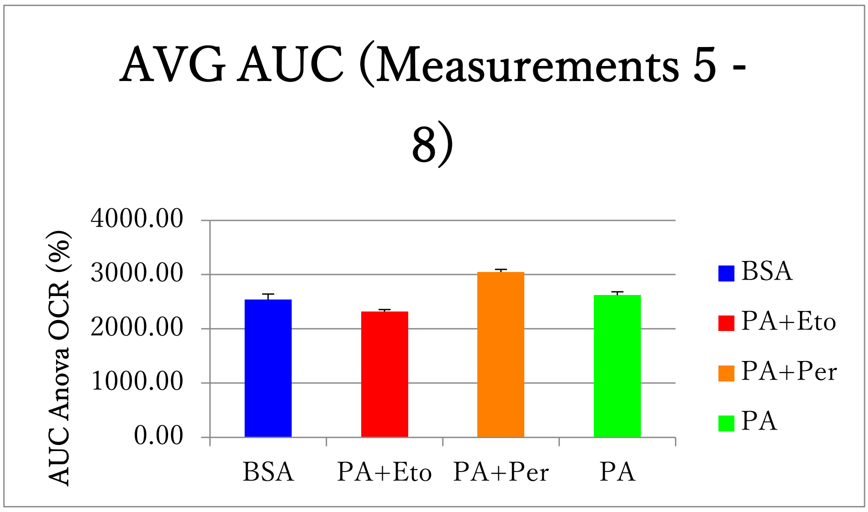

P Value

(Tukey Post test)

	BSA	PA+Eto	PA+Per	PA
BSA		0.001305	0.000000	0.247085
PA+Eto			0.000000	0.000045
PA+Per				0.000000

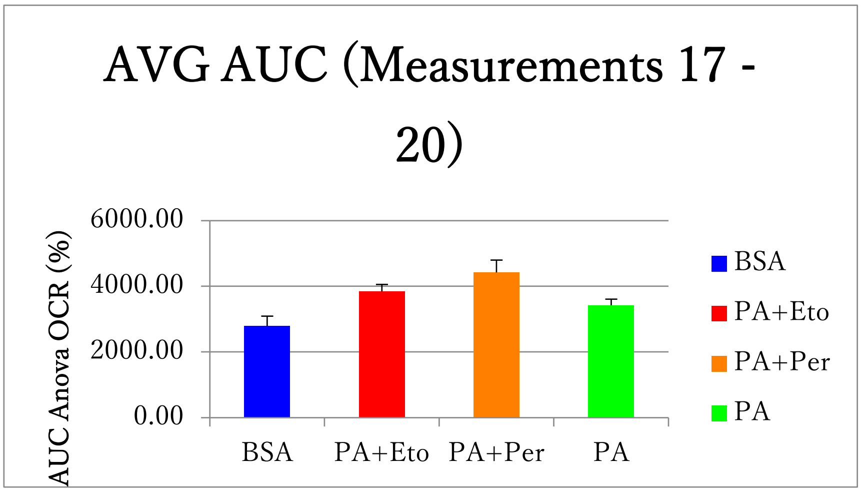

P Value

(Tukey Post test)

	BSA	PA+Eto	PA+Per	PA
BSA		0.000309	0.000001	0.014719
PA+Eto			0.035083	0.167713
PA+Per				0.000270

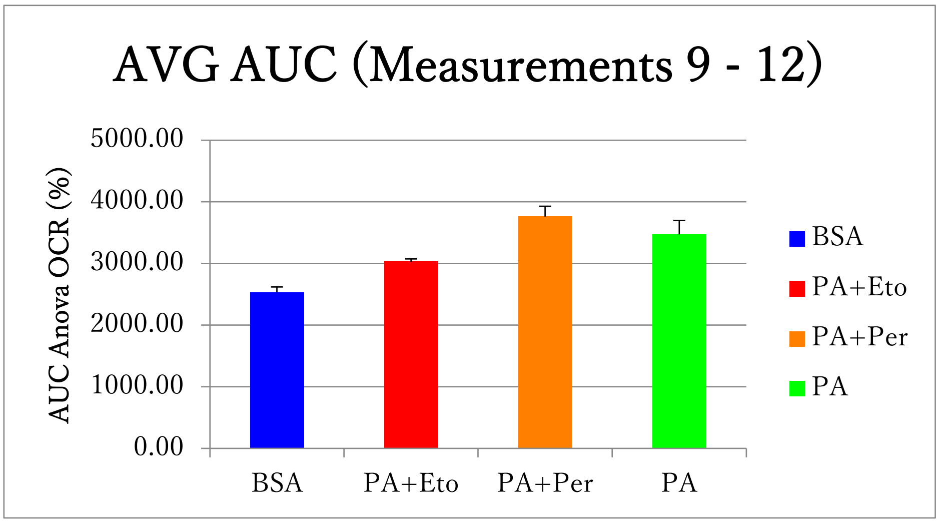

P Value

(Tukey Post test)

	BSA	PA+Eto	PA+Per	PA
BSA		0.001071	0.000000	0.000000
PA+Eto			0.000020	0.003231
PA+Per				0.042957

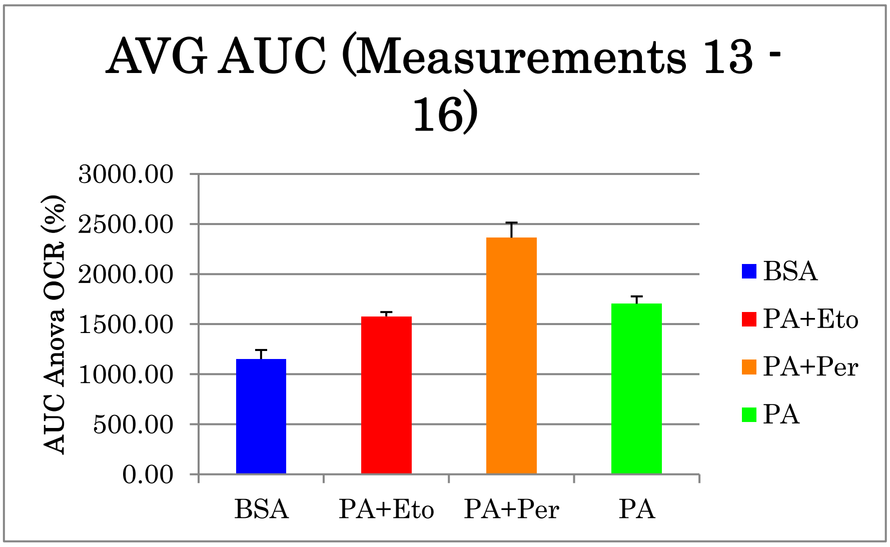

P Value

(Tukey Post test)

	BSA	PA+Eto	PA+Per	PA
BSA		0.000070	0.000000	0.000002
PA+Eto			0.000000	0.285068
PA+Per				0.000000

B

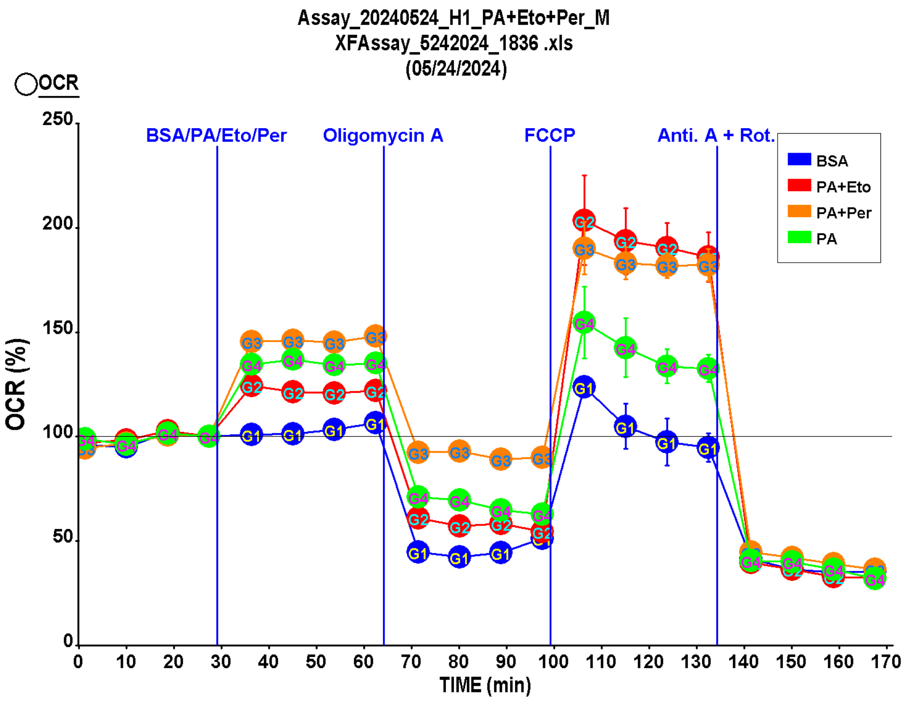

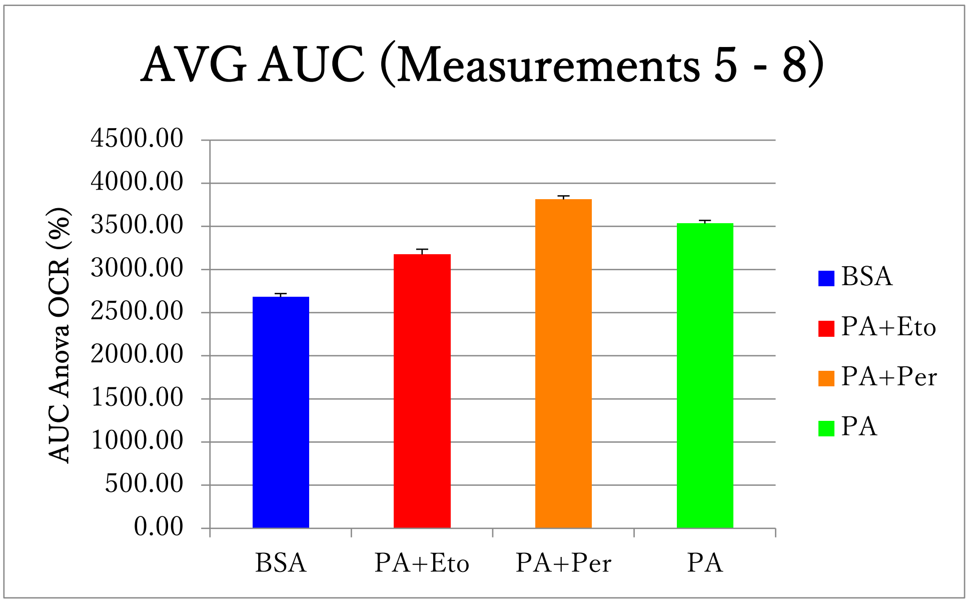

P Value (Tukey Post test)

	BSA	PA+Eto	PA+Per	PA
BSA		0.000000	0.000000	0.000000
PA+Eto			0.000000	0.000000
PA+Per				0.000000

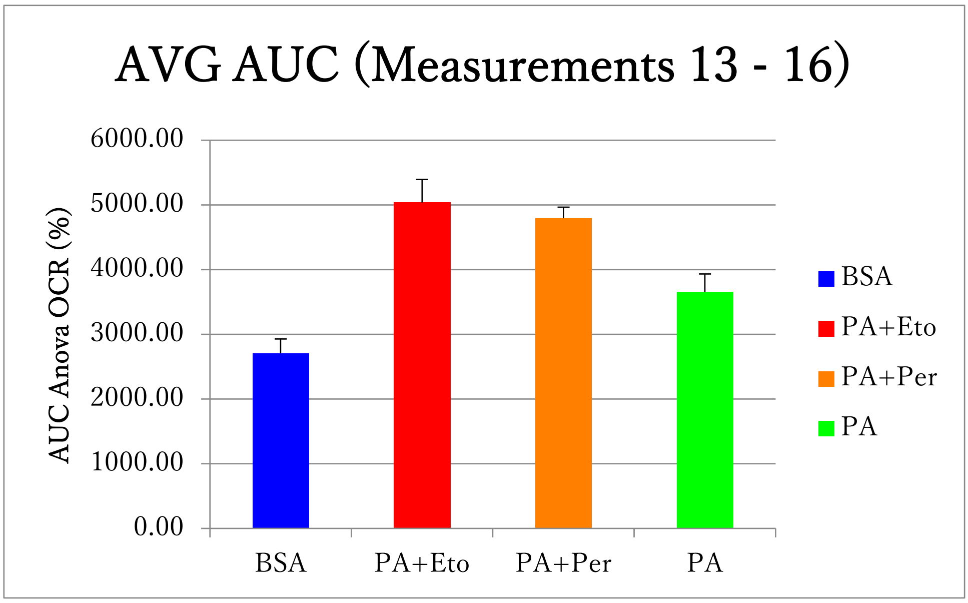

P Value (Tukey Post test)

	BSA	PA+Eto	PA+Per	PA

BSA		0.000000	0.000000	0.000204
PA+Eto			0.496578	0.000002
PA+Per				0.000024

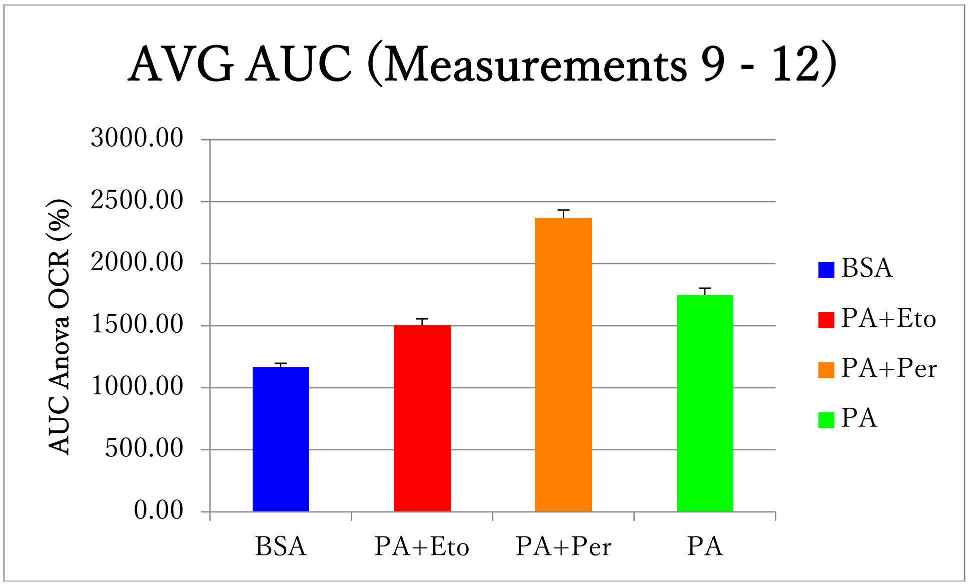

P Value (Tukey Post test)

	BSA	PA+Eto	PA+Per	PA
BSA		0.000000	0.000000	0.000000
PA+Eto			0.000000	0.000008
PA+Per				0.000000
