# Supplementary material for: Rates of Mitochondrial Metabolism of Glucose, Amino Acids, and Fatty Acids by the HEI-OC1 Inner Ear Cell Line
Source: Biology (Basel). 2025 Aug 24;14(9):1118. doi: 10.3390/biology14091118 (PMC12467209; doi:10.3390/biology14091118)
Supplement: Supplementary file 1 [file biology-14-01118-s001.zip › Suppl.S2 Statistical Analysis/Statistical Analysis Results(Fig.2).pdf]

AVG AUC (X–Y)" refers to the average oxygen consumption rate calculated from timepoints X to Y during the plateau phase after substrate or inhibitor addition.

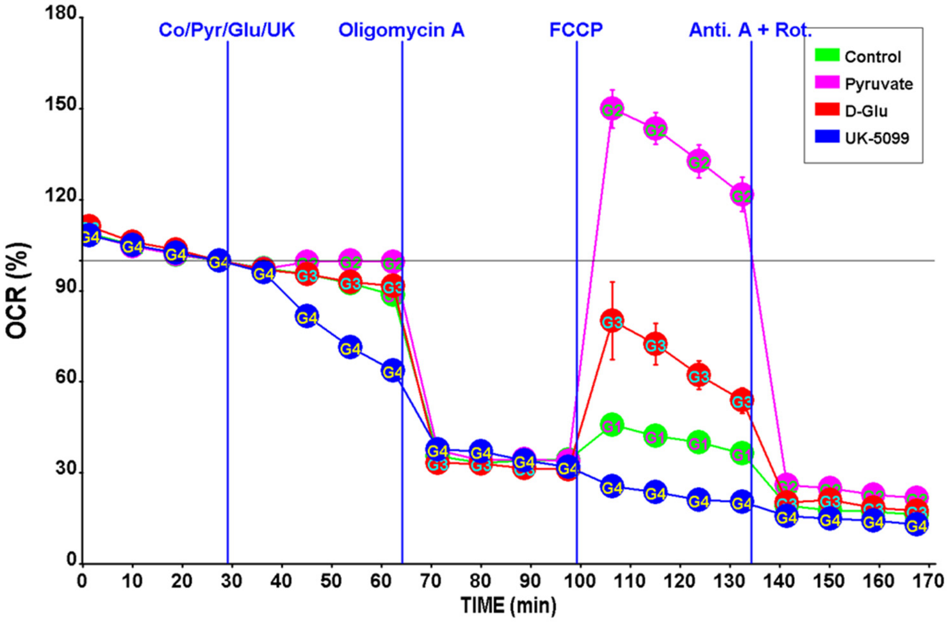

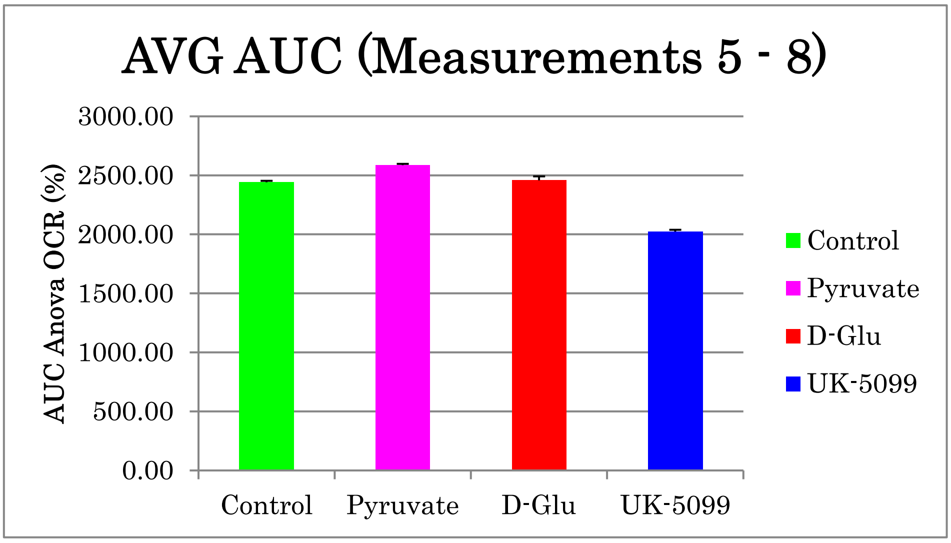

P Value

(Tukey Post test)

	Control	Pyruvate	D-Glu	UK-5099
Control		0.000000	0.572277	0.000000
Pyruvate			0.000000	0.000000
D-Glu				0.000000
UK-5099				

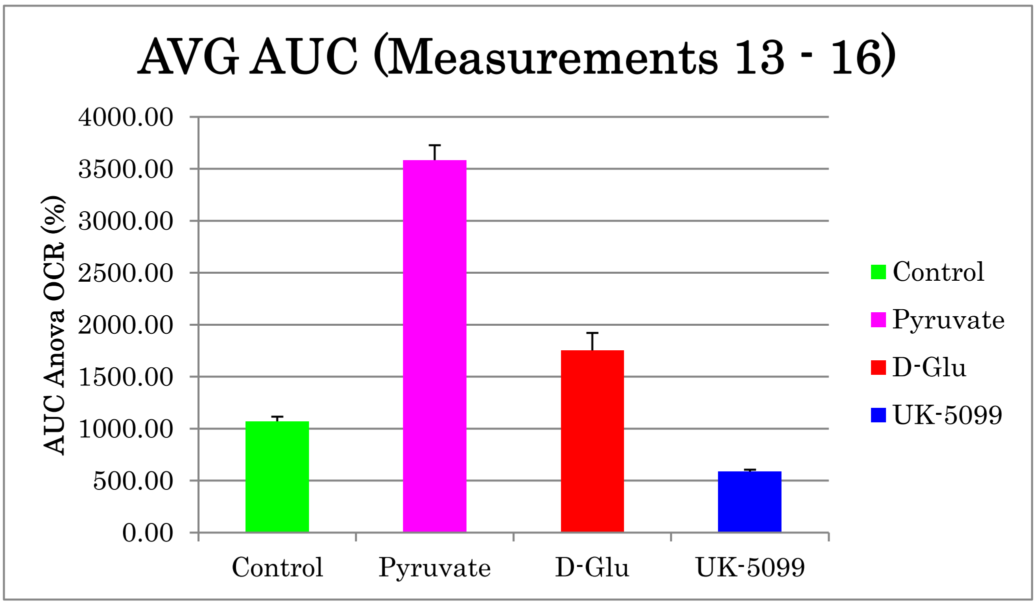

PValue

(Tukey Post test)

	Control	Pyruvate	D-Glu	UK-5099
Control		0.000000	0.000000	0.000024
Pyruvate			0.000000	0.000000
D-Glu				0.000000
UK-5099				
