# Supplementary material for: Rates of Mitochondrial Metabolism of Glucose, Amino Acids, and Fatty Acids by the HEI-OC1 Inner Ear Cell Line
Source: Biology (Basel). 2025 Aug 24;14(9):1118. doi: 10.3390/biology14091118 (PMC12467209; doi:10.3390/biology14091118)
Supplement: Supplementary file 1 [file biology-14-01118-s001.zip › Suppl.S2 Statistical Analysis/Statistical Analysis Results(Fig.3).pdf]

AVG AUC (X–Y)" refers to the average oxygen consumption rate calculated from timepoints X to Y during the plateau phase after substrate or inhibitor addition.

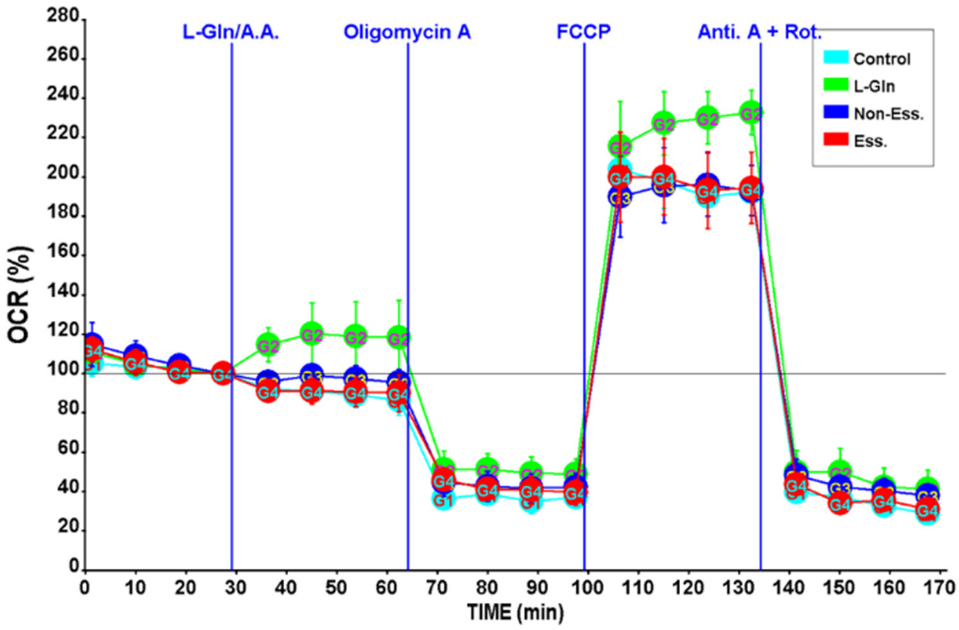

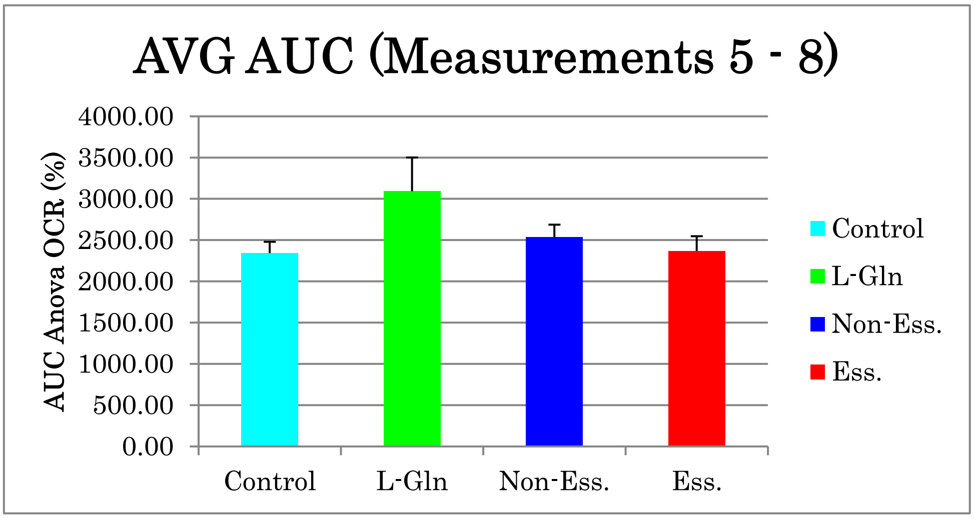

P Value

(Tukey Post test)

	Control	L-Gln	Non-Ess.	Ess.
Control		0.001769	0.626296	0.999055
L-Gln			0.017605	0.007430
Non-Ess.				0.795512

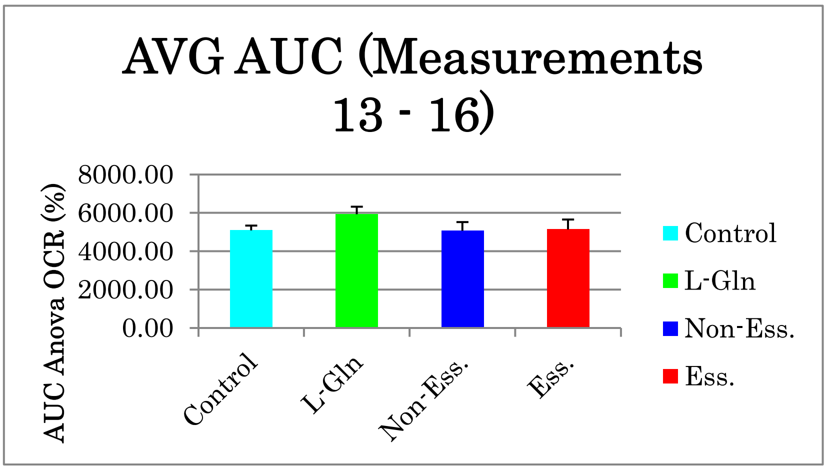

P Value (Tukey Post test)

	Control	L-Gln	Non-Ess.	Ess.
Control		0.022665	0.999838	0.998733
L-Gln			0.019652	0.068293
Non-Ess.				0.996292

B

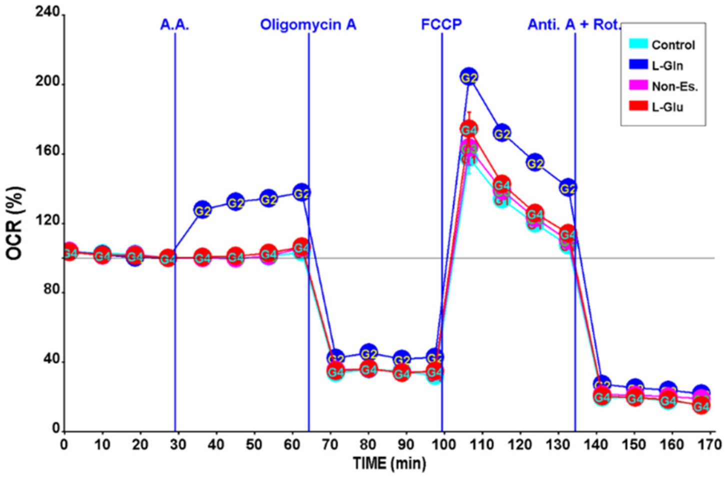

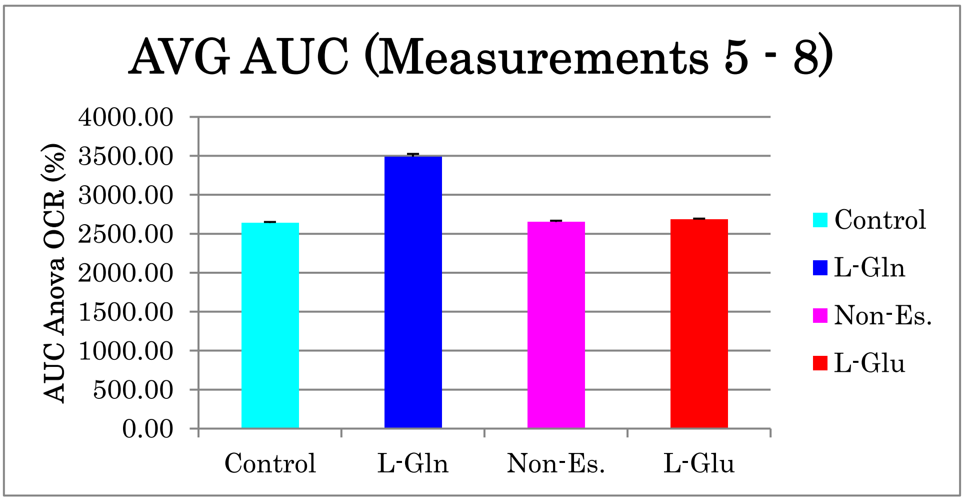

P Value

(Tukey Post test)

	Control	L-Gln	Non-Es.	L-Glu
Control		0.000000	0.933868	0.953275
L-Gln			0.000000	0.000000
Non-Es.				0.999870

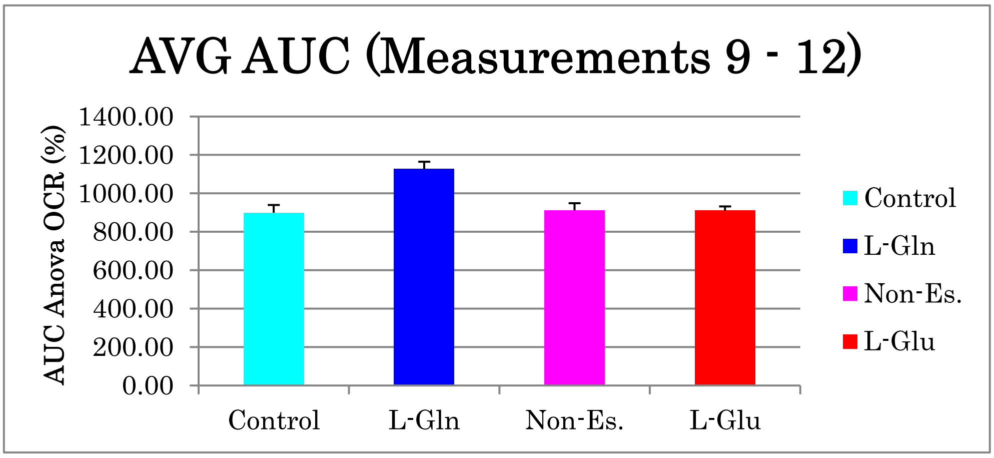

P Value

(Tukey Post test)

	Control	L-Gln	Non-Es.	L-Glu
Control		0.000000	0.774770	0.017486
L-Gln			0.000000	0.000000
Non-Es.				0.109770

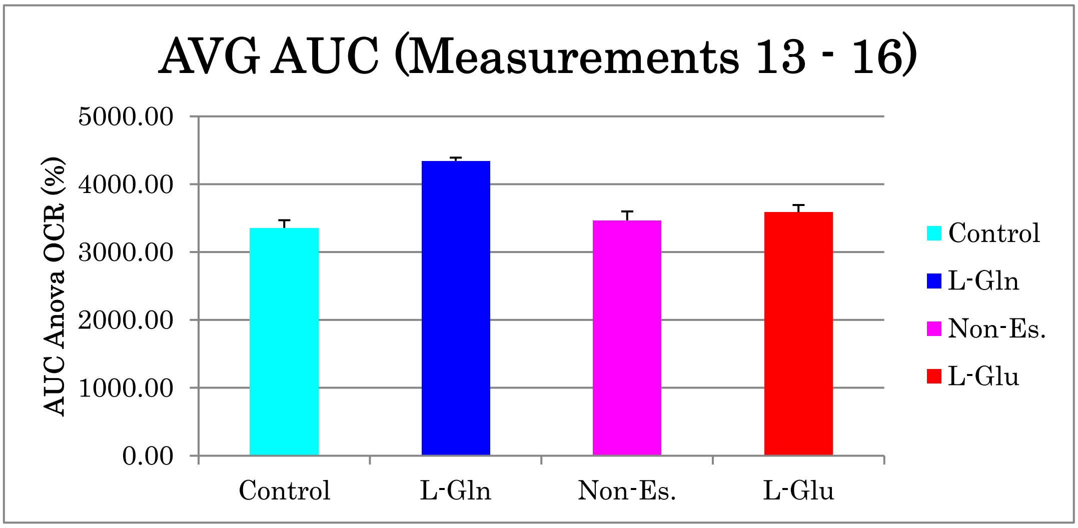

P Value

(Tukey Post test)

	Control	L-Gln	Non-Es.	L-Glu
Control		0.000000	0.363516	0.014096
L-Gln			0.000000	0.000000
Non-Es.				0.301355
