# Supplementary material for: Rates of Mitochondrial Metabolism of Glucose, Amino Acids, and Fatty Acids by the HEI-OC1 Inner Ear Cell Line
Source: Biology (Basel). 2025 Aug 24;14(9):1118. doi: 10.3390/biology14091118 (PMC12467209; doi:10.3390/biology14091118)
Supplement: Supplementary file 1 [file biology-14-01118-s001.zip › Suppl.S2 Statistical Analysis/Statistical Analysis Results(Fig.4).pdf]

AVG AUC (X–Y)" refers to the average oxygen consumption rate calculated from timepoints X to Y during the plateau phase after substrate or inhibitor addition.

A

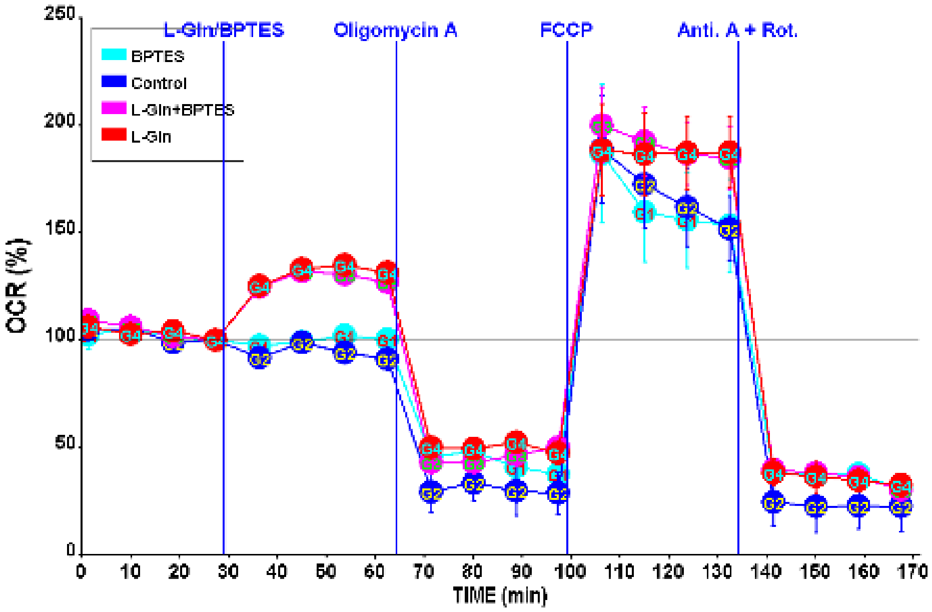

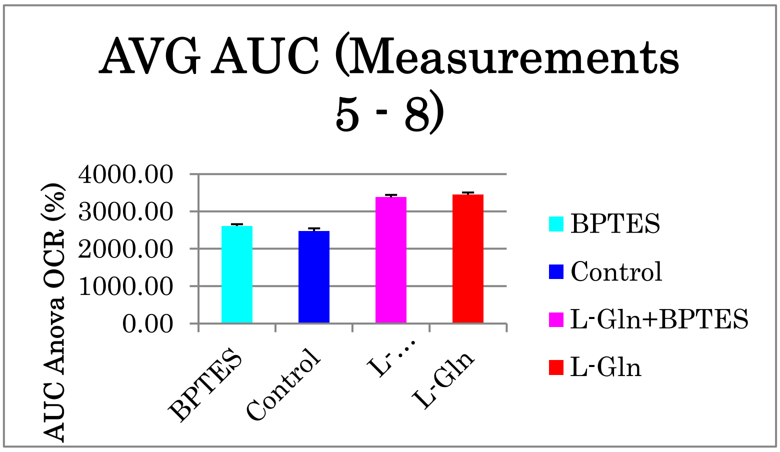

P Value (Tukey Post test)

	BPTES	Control	L-Gln+BPTES	L-Gln
BPTES		0.013515	0.000000	0.000000
Control			0.000000	0.000000
L-Gln+BPTES				0.277902

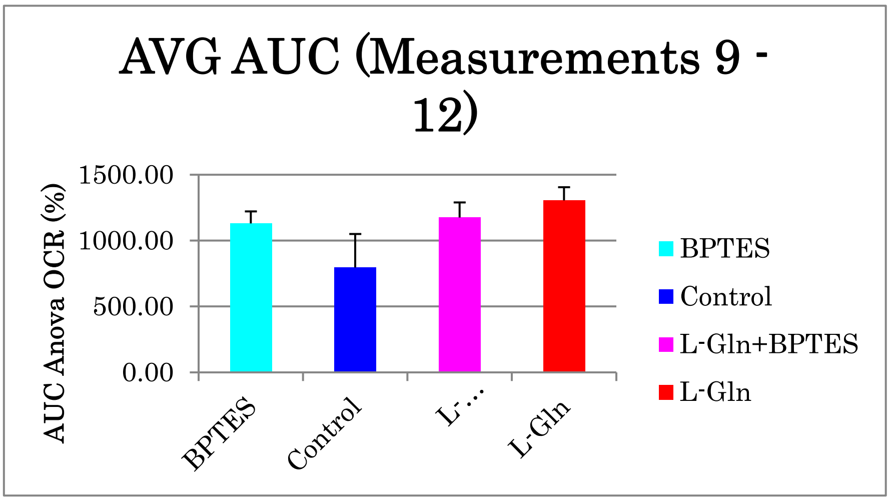

P Value

(Tukey Post test)

	BPTES	Control	L-Gln+BPTES	L-Gln
BPTES		0.016715	0.968244	0.316373
Control			0.006687	0.000459
L-Gln+BPTES				0.557226

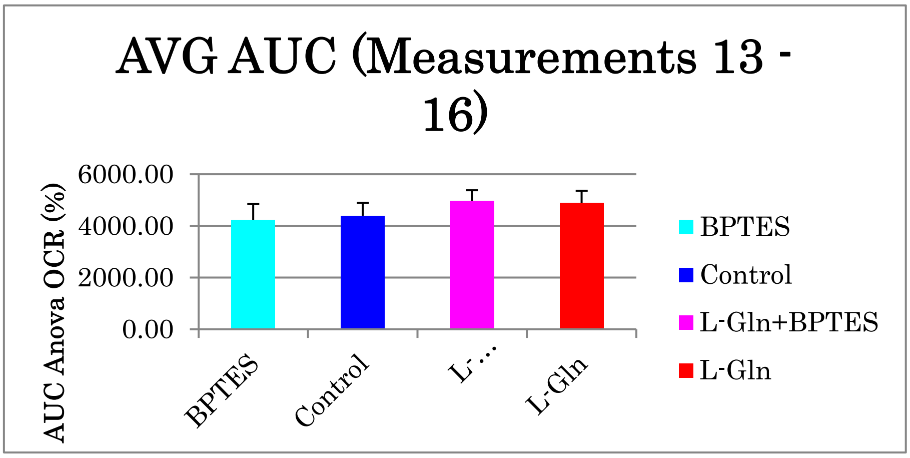

P Value (Tukey

Post test)

	BPTES	Control	L-Gln+BPTES	L-Gln
BPTES		0.953086	0.133148	0.210613
Control			0.305851	0.442282
L-Gln+BPTES				0.992461

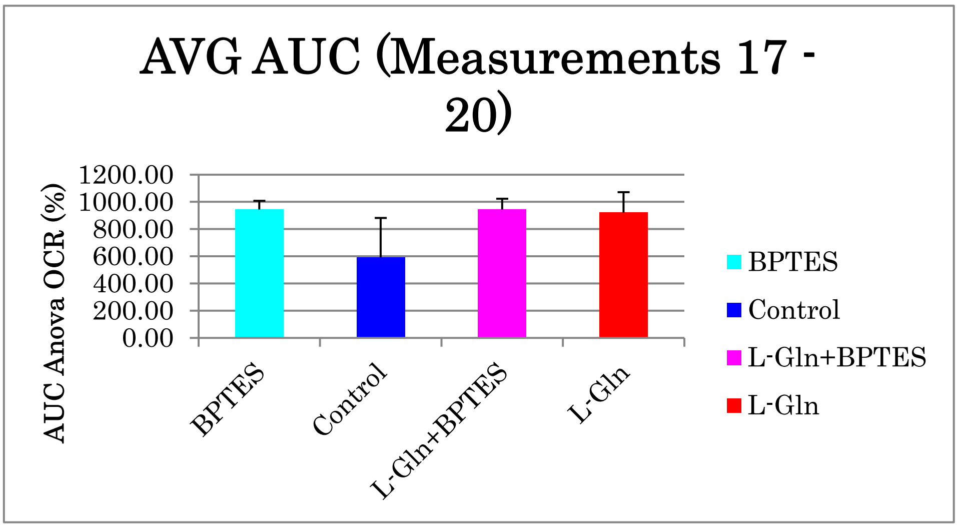

P Value

(Tukey Post test)

	BPTES	Control	L-Gln+BPTES	L-Gln
BPTES		0.023177	1.000000	0.996678
Control			0.022850	0.034962
L-Gln+BPTES				0.996330

B

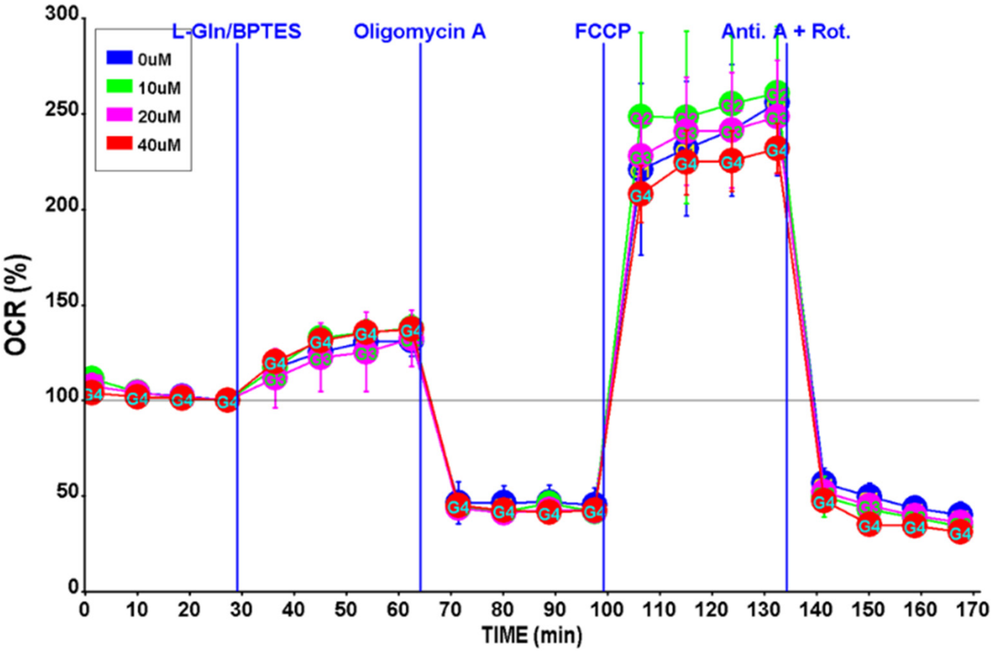

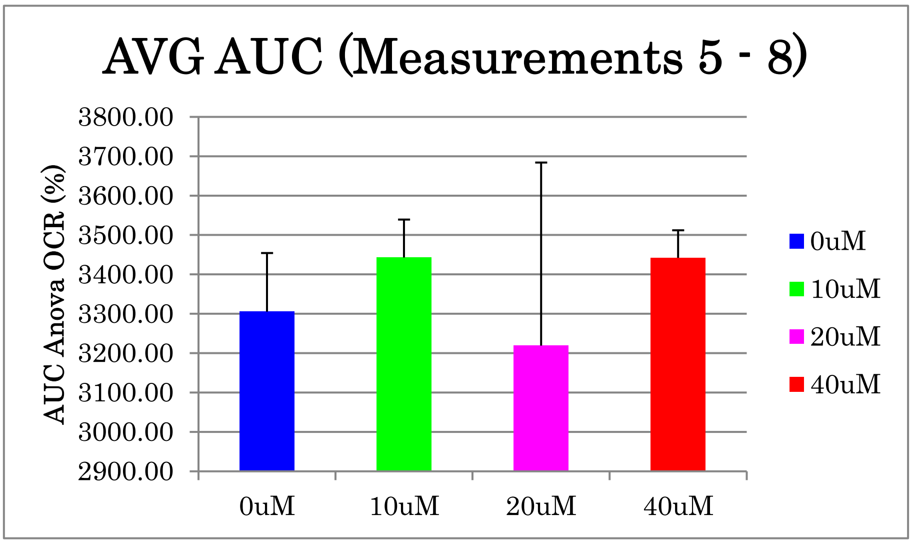

P Value

(Tukey Post test)

	0uM	10uM	20uM	40uM
0uM		0.712387	0.935970	0.717129
10uM			0.458584	1.000000
20uM				0.462532

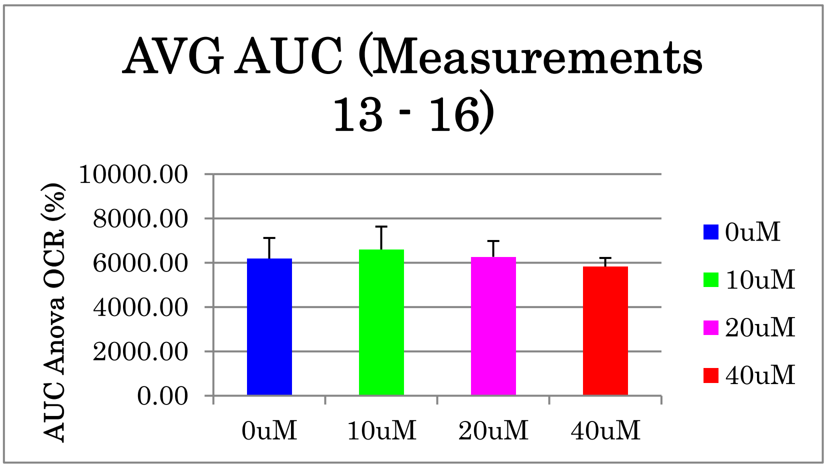

P Value

(Tukey Post test)

	0uM	10uM	20uM	40uM
0uM		0.858883	0.999230	0.894585
10uM			0.943652	0.469014
20uM				0.881384
