# Supplementary material for: Rates of Mitochondrial Metabolism of Glucose, Amino Acids, and Fatty Acids by the HEI-OC1 Inner Ear Cell Line
Source: Biology (Basel). 2025 Aug 24;14(9):1118. doi: 10.3390/biology14091118 (PMC12467209; doi:10.3390/biology14091118)
Supplement: Supplementary file 1 [file biology-14-01118-s001.zip › Suppl.S2 Statistical Analysis/Statistical Analysis Results(Fig.5).pdf]

AVG AUC (X–Y)" refers to the average oxygen consumption rate calculated from timepoints X to Y during the plateau phase after substrate or inhibitor addition.

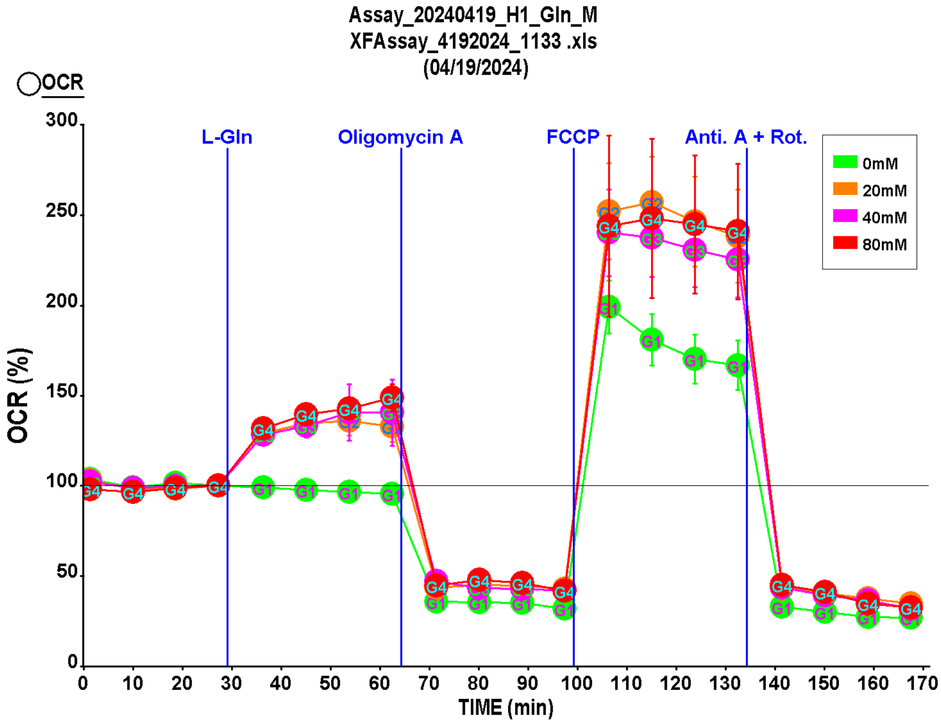

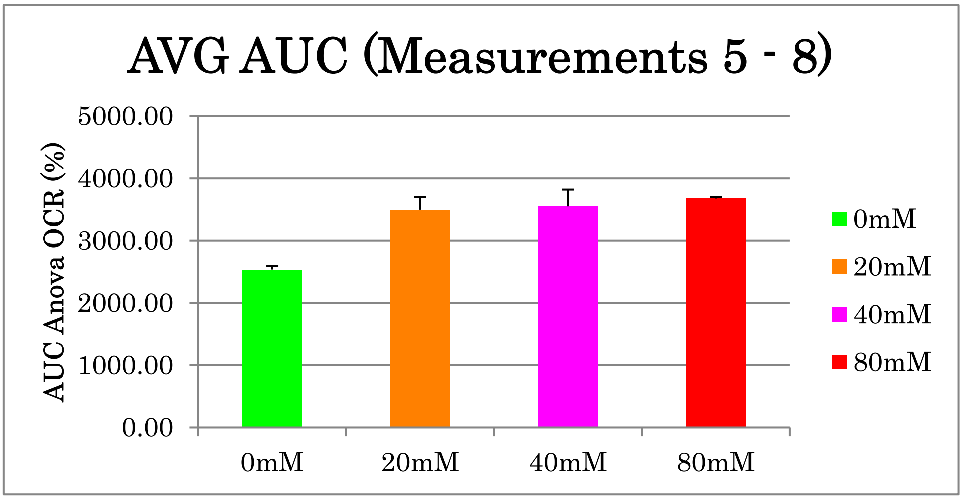

P Value

(Tukey Post test)

	0mM	20mM	40mM	80mM
0mM		0.000004	0.000002	0.000003
20mM			0.965187	0.549100
40mM				0.781669

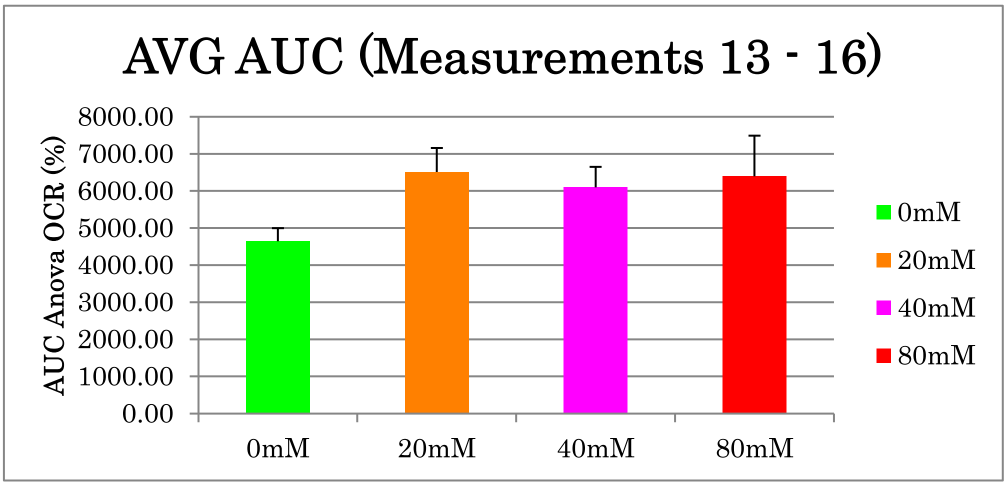

P Value

(Tukey Post test)

	0mM	20mM	40mM	80mM
0mM		0.002146	0.014564	0.010771
20mM			0.744191	0.994805
40mM				0.918490

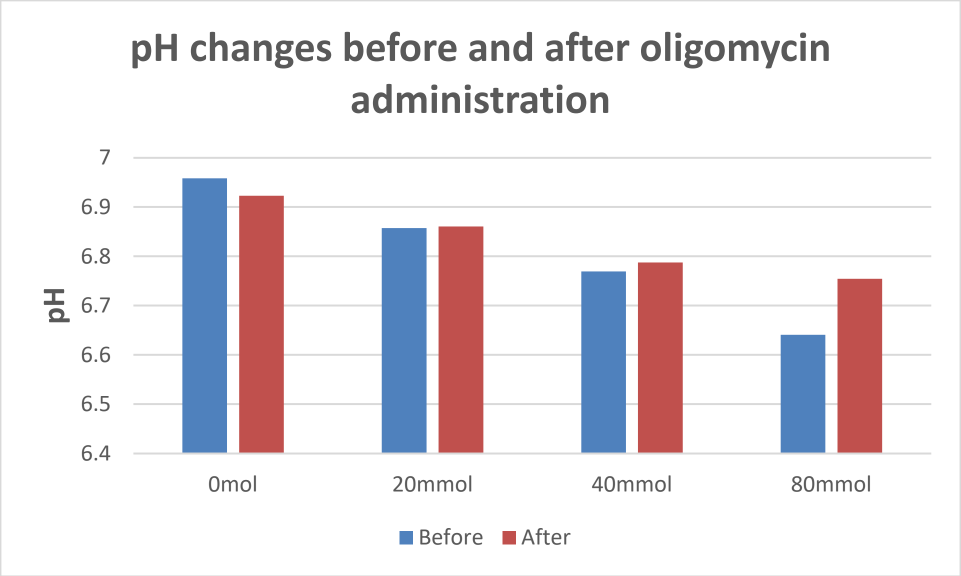

	**Before**	**After**	**p value**
**0mol**	**6.958427313**	**6.922839074**	**0.175184034**
**20mmol**	**6.857223188**	**6.860489762**	**0.938635673**
**40mmol**	**6.769320229**	**6.787536197**	**0.276092538**
**80mmol**	**6.640784681**	**6.754302716**	**0.006482254**

B

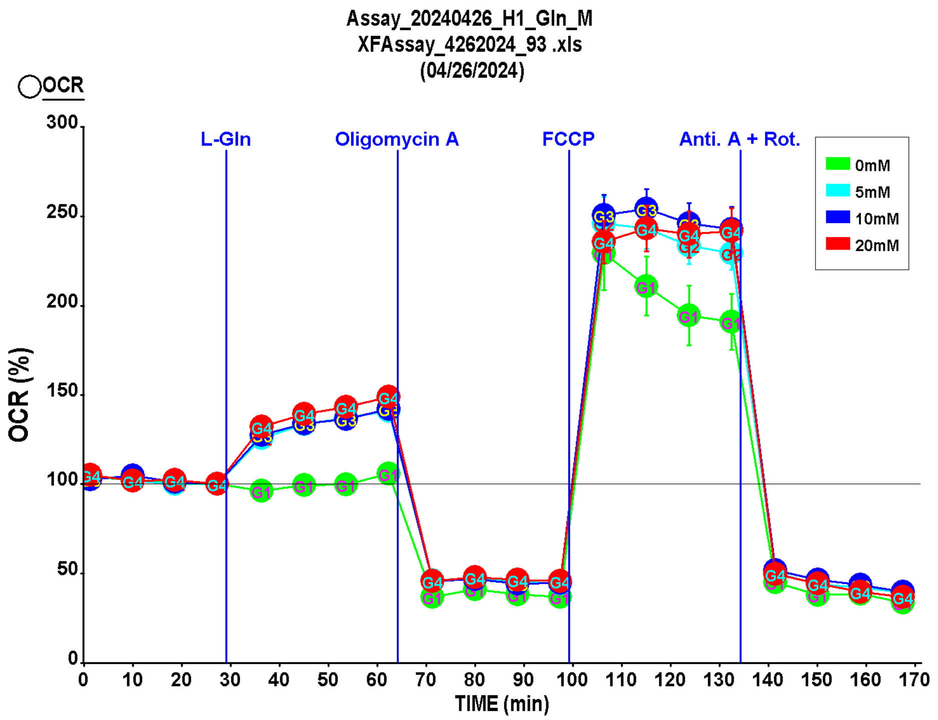

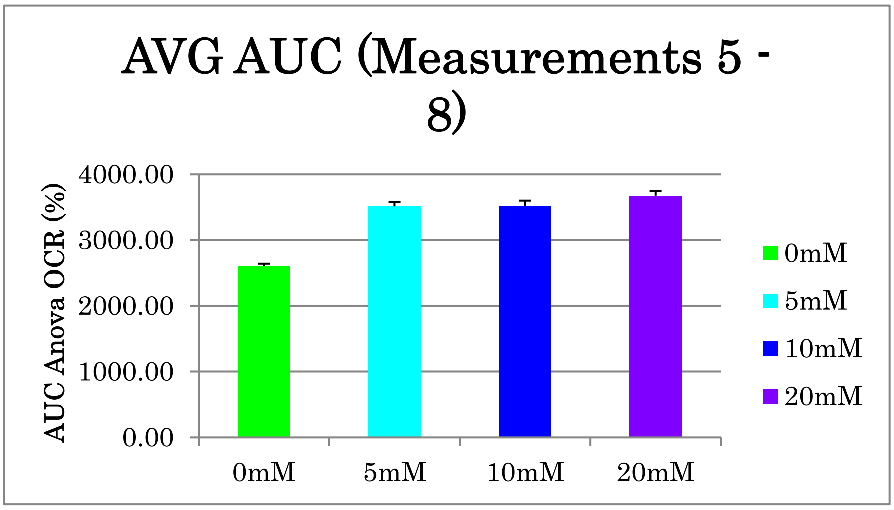

P Value

(Tukey Post test)

	0mM	5mM	10mM	20mM
0mM		0.000000	0.000000	0.000000
5mM			0.997184	0.005412
10mM				0.008079

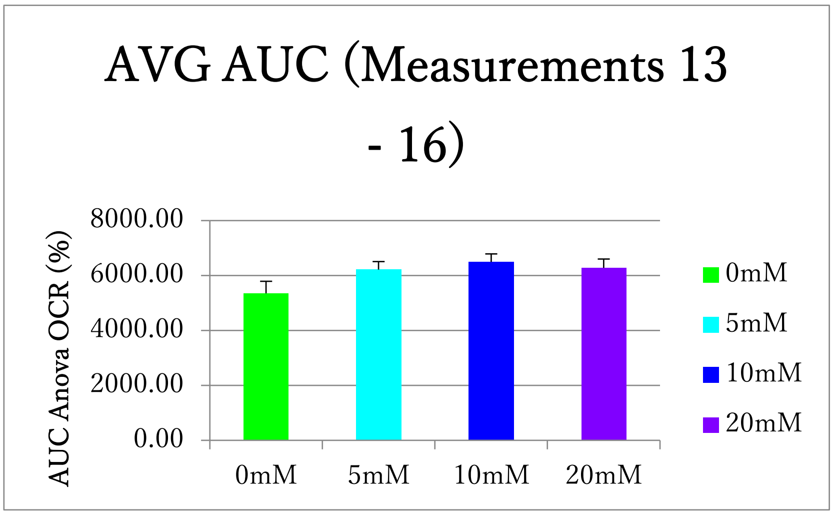

P Value

(Tukey Post test)

	0mM	5mM	10mM	20mM
0mM		0.005346	0.000394	0.002946
5mM			0.576220	0.990914
10mM				0.747806

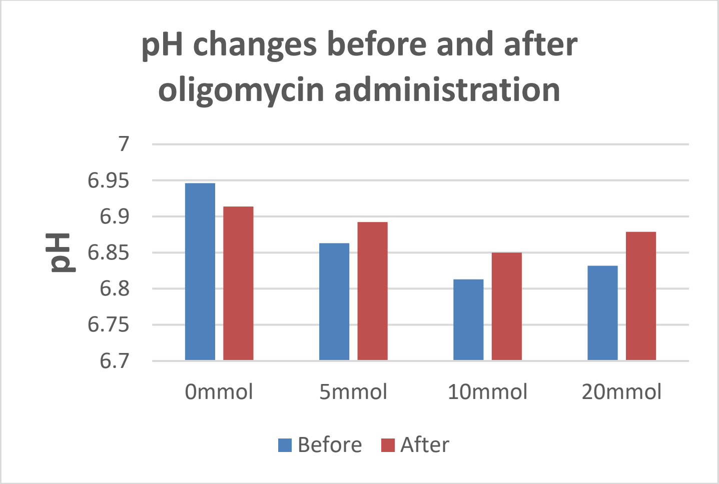

	**Before**	**After**	**p value**
**0mmol**	**6.945987382**	**6.91379773**	**0.287854257**
**5mmol**	**6.863006026**	**6.892235081**	**0.489622027**
**10mmol**	**6.812975813**	**6.849832125**	**0.446770411**
**20mmol**	**6.831706334**	**6.878773327**	**0.049309087**

C

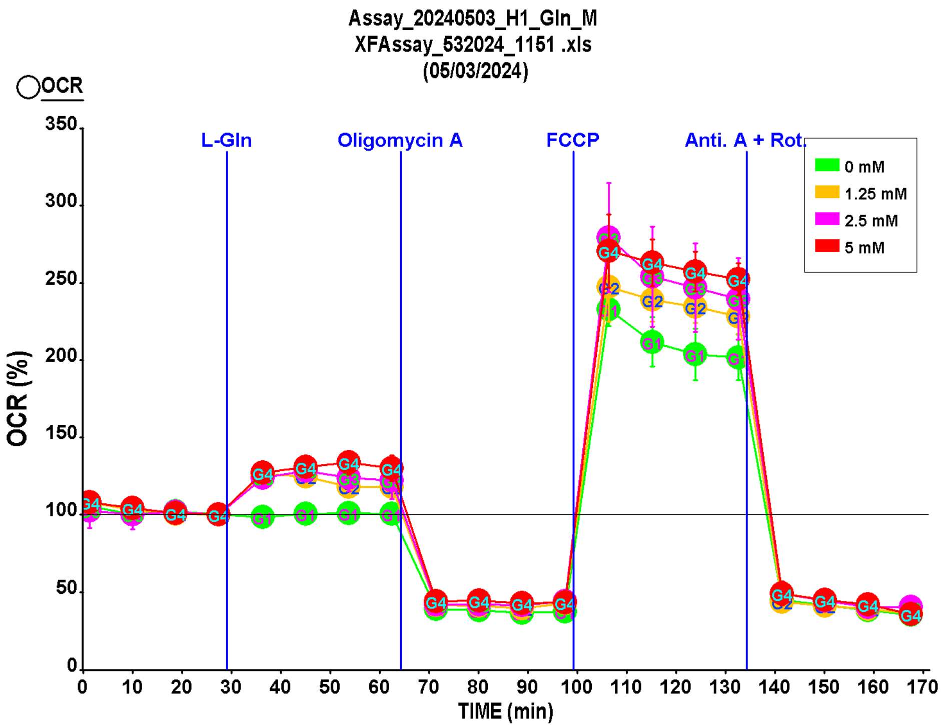

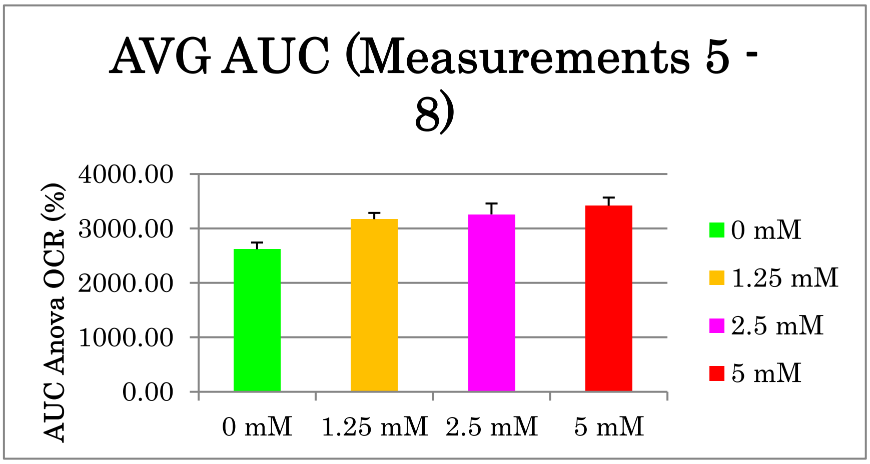

P Value

(Tukey Post test)

	0 mM	1.25 mM	2.5 mM	5 mM
0 mM		0.000150	0.000026	0.000002
1.25 mM			0.781179	0.081849
2.5 mM				0.378738

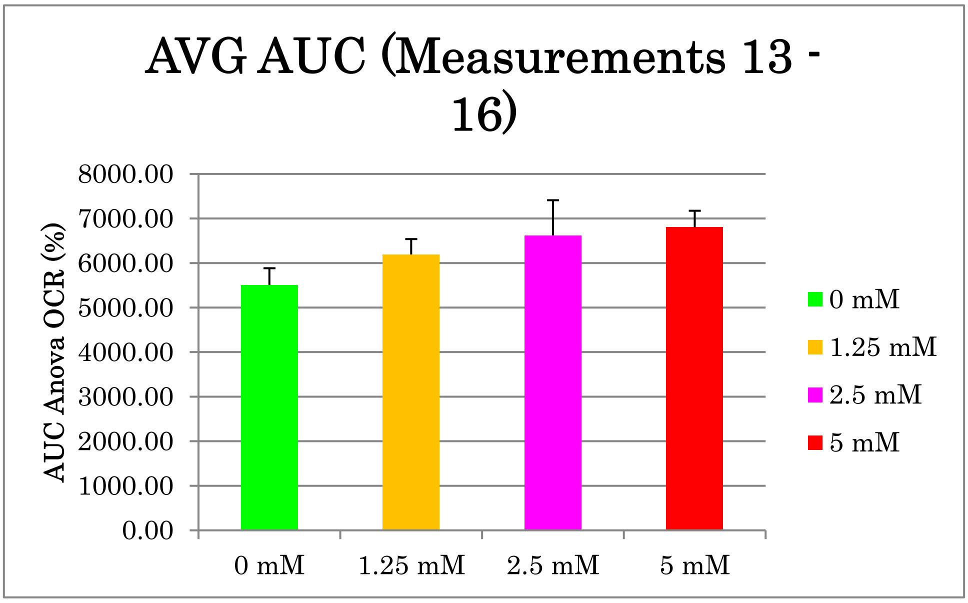

P Value

(Tukey Post test)

	0 mM	1.25 mM	2.5 mM	5 mM
0 mM		0.181992	0.014822	0.004603
1.25 mM			0.555755	0.262836
2.5 mM				0.937881

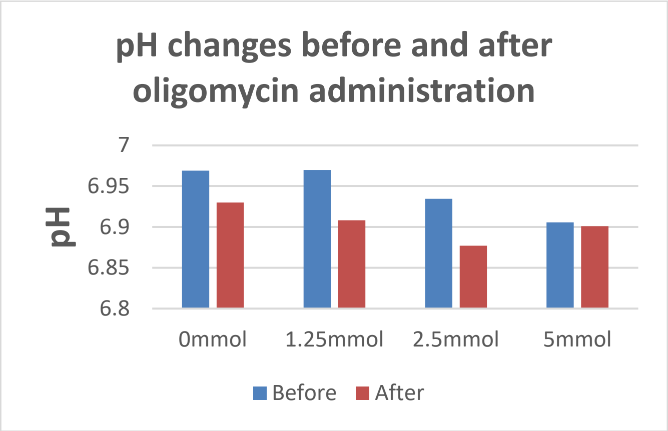

	**Before**	**After**	**P value**
**0mmol**	**6.968794565**	**6.929938455**	**0.124533252**
**1.25mmol**	**6.969719002**	**6.908088756**	**0.163190747**
**2.5mmol**	**6.934327216**	**6.877015015**	**0.06**
**5mmol**	**6.9055331**	**6.900835469**	**0.870872917**
