# Supplementary material for: Rates of Mitochondrial Metabolism of Glucose, Amino Acids, and Fatty Acids by the HEI-OC1 Inner Ear Cell Line
Source: Biology (Basel). 2025 Aug 24;14(9):1118. doi: 10.3390/biology14091118 (PMC12467209; doi:10.3390/biology14091118)
Supplement: Supplementary file 1 [file biology-14-01118-s001.zip › Suppl.S2 Statistical Analysis/Statistical Analysis Results(Fig.6).pdf]

AVG AUC (X–Y)" refers to the average oxygen consumption rate calculated from timepoints X to Y during the plateau phase after substrate or inhibitor addition.

A :

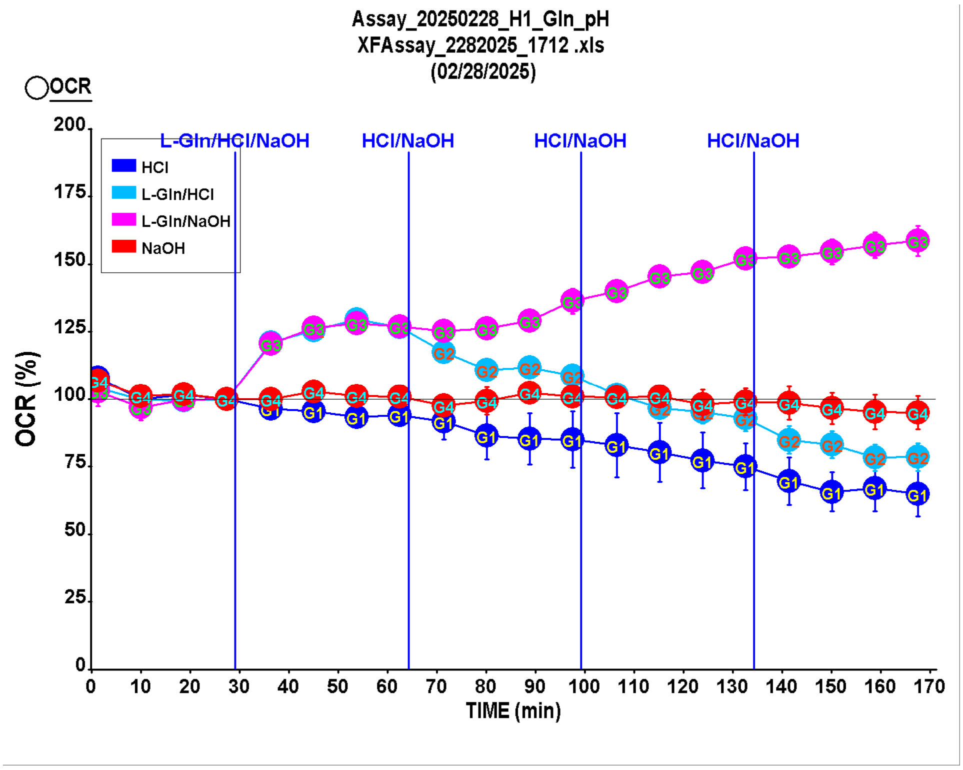


B:

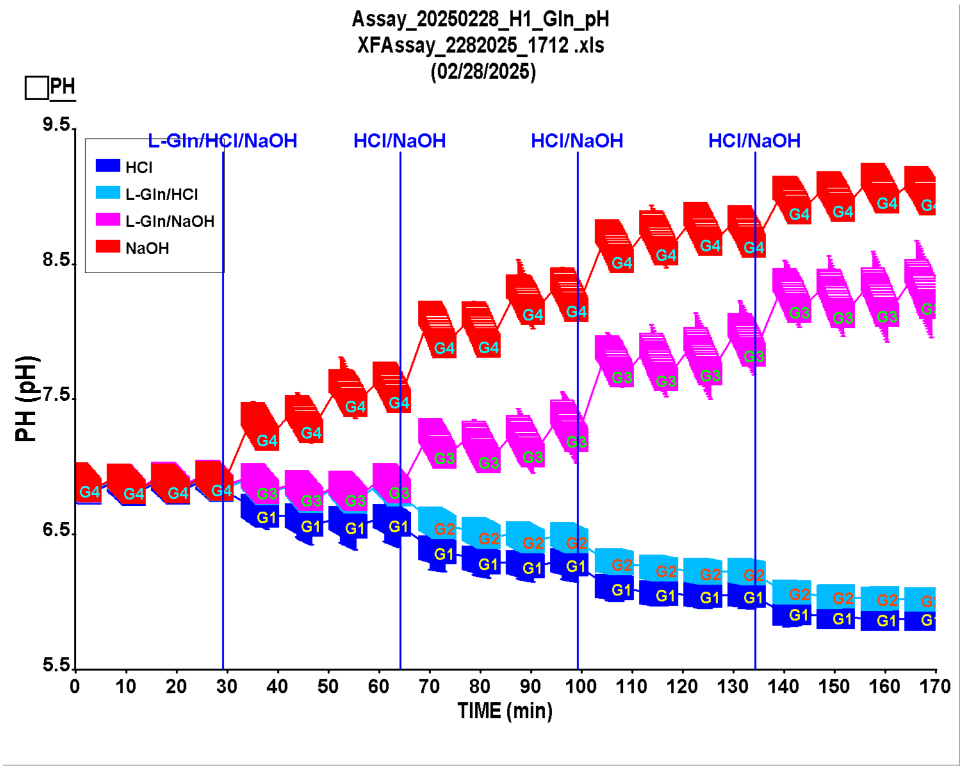

C:

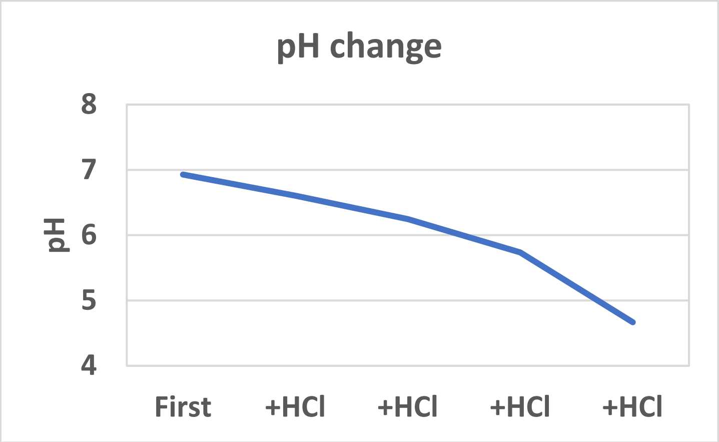


| | Average | STDEV |
|---|---|---|
| First | 6.93 | 0.021602 |
| +HCl | 6.603333 | 0.018856 |
| +HCl | 6.246667 | 0.016997 |
| +HCl | 5.736667 | 0.012472 |
| +HCl | 4.666667 | 0.009428 |

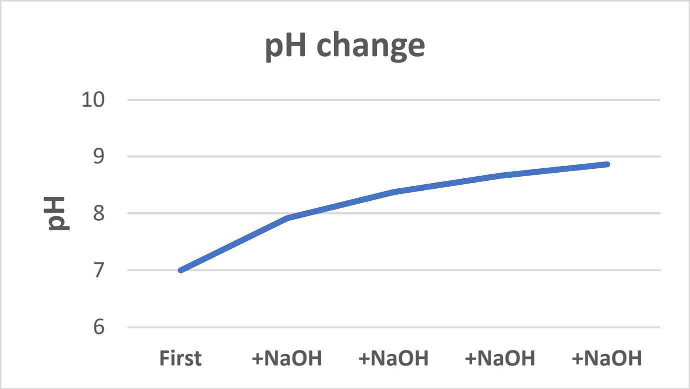


| | Average | STDEV |
|---|---|---|
| First | 7 | 0.021602 |
| +NaOH | 7.916667 | 0.018856 |
| +NaOH | 8.376667 | 0.016997 |
| +NaOH | 8.663333 | 0.012472 |
| +NaOH | 8.863333 | 0.009428 |

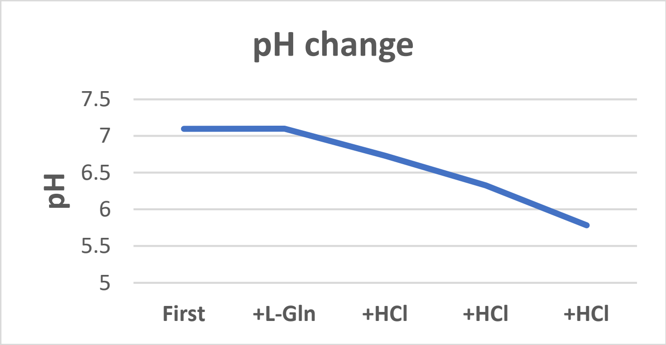


| | Average | STDEV |
|---|---|---|
| First | 7.096667 | 0.021602 |
| +L-Gln | 7.1 | 0.018856 |
| +HCl | 6.73 | 0.016997 |
| +HCl | 6.326667 | 0.012472 |
| +HCl | 5.783333 | 0.009428 |

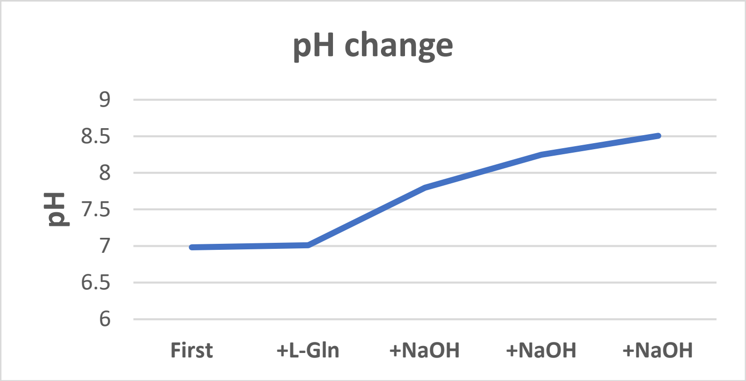


| | Average | STDEV |
|---|---|---|
| First | 6.983333 | 0.021602 |
| +L-Gln | 7.01 | 0.018856 |
| +NaOH | 7.796667 | 0.016997 |
| +NaOH | 8.246667 | 0.012472 |
| +NaOH | 8.506667 | 0.009428 |

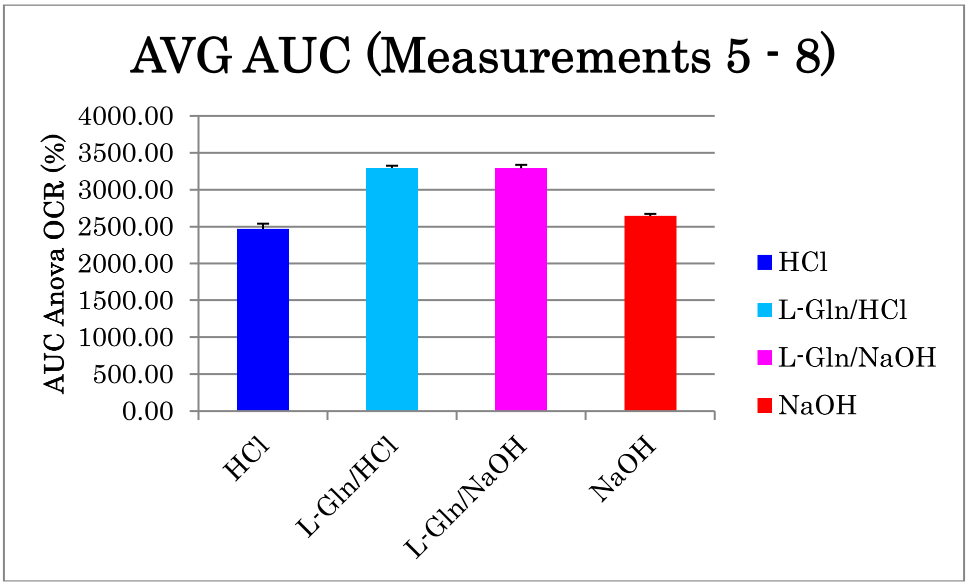


**P Value (Tukey Post test)**

| | HCl | L-Gln/HCl | L-Gln/NaOH | NaOH |
|---|---|---|---|---|
| HCl | | 0.000000 | 0.000000 | 0.000132 |
| L-Gln/HCl | | | 0.997805 | 0.000000 |
| L-Gln/NaOH | | | | 0.000000 |
| NaOH | | | | |

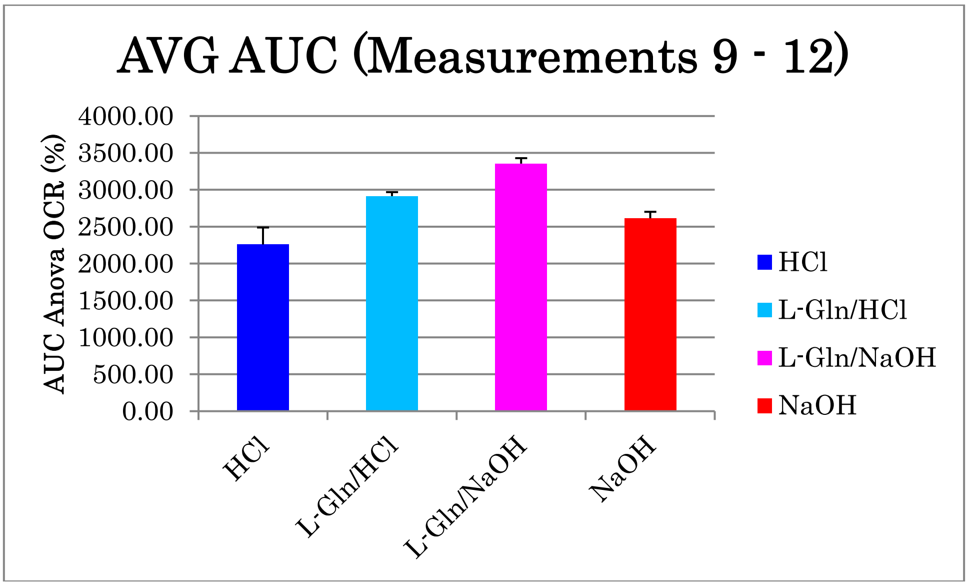


**P Value (Tukey Post test)**

| | HCl | L-Gln/HCl | L-Gln/NaOH | NaOH |
|---|---|---|---|---|
| HCl | | 0.000004 | 0.000000 | 0.002975 |
| L-Gln/HCl | | | 0.000393 | 0.011361 |
| L-Gln/NaOH | | | | 0.000001 |
| NaOH | | | | |

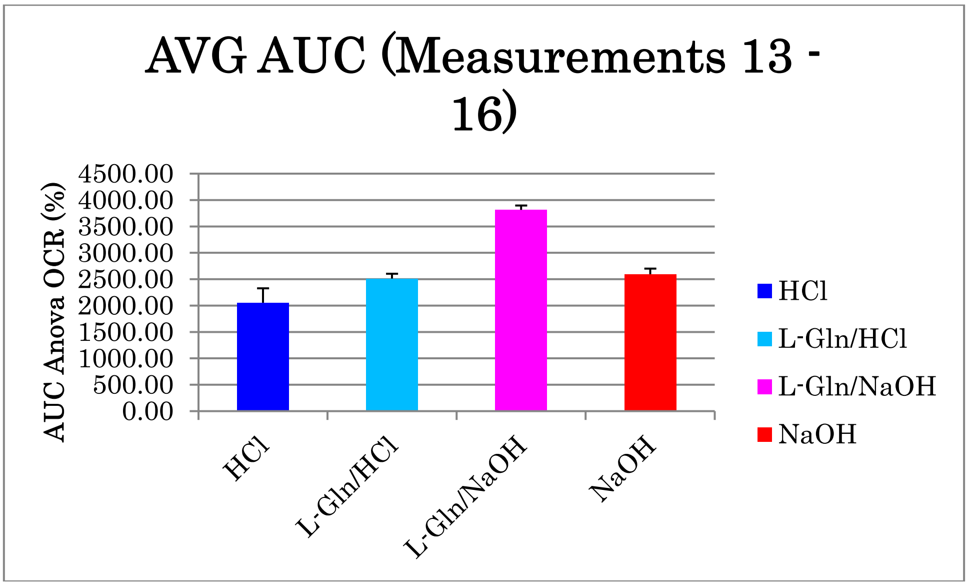


**P Value (Tukey Post test)**

| | HCl | L-Gln/HCl | L-Gln/NaOH | NaOH |
|---|---|---|---|---|
| HCl | | 0.001688 | 0.000000 | 0.000335 |
| L-Gln/HCl | | | 0.000000 | 0.846575 |
| L-Gln/NaOH | | | | 0.000000 |
| NaOH | | | | |

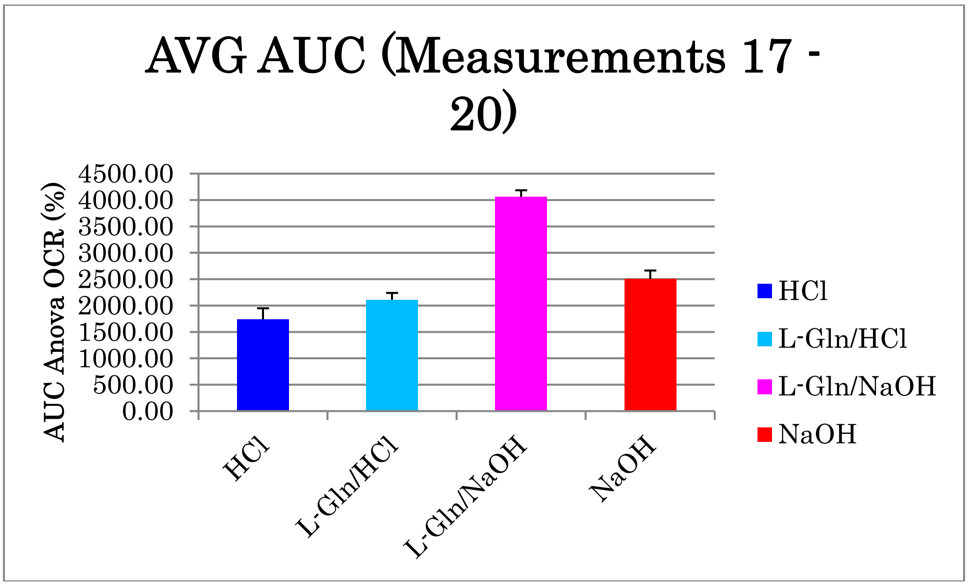


**P Value (Tukey Post test)**

| | HCl | L-Gln/HCl | L-Gln/NaOH | NaOH |
|---|---|---|---|---|
| HCl | | 0.008528 | 0.000000 | 0.000005 |
| L-Gln/HCl | | | 0.000000 | 0.005570 |
| L-Gln/NaOH | | | | 0.000000 |
| NaOH | | | | |
