# Supplementary material for: Rates of Mitochondrial Metabolism of Glucose, Amino Acids, and Fatty Acids by the HEI-OC1 Inner Ear Cell Line
Source: Biology (Basel). 2025 Aug 24;14(9):1118. doi: 10.3390/biology14091118 (PMC12467209; doi:10.3390/biology14091118)
Supplement: Supplementary file 1 [file biology-14-01118-s001.zip › Suppl.S2 Statistical Analysis/Statistical Analysis Results(Fig.8 ).pdf]

AVG AUC (X–Y)" refers to the average oxygen consumption rate calculated from timepoints X to Y during the plateau phase after substrate or inhibitor addition.

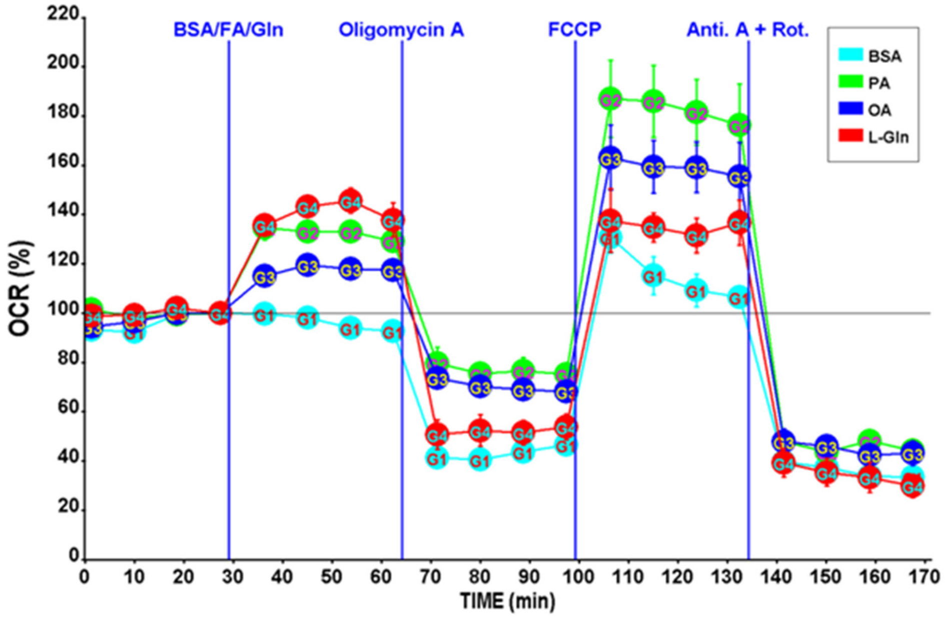

Abbreviation; BSA: Bovine serum albumin PA: Palmitic acid (saturated fatty acid) OA: Oleic acid (unsaturated fatty acid)

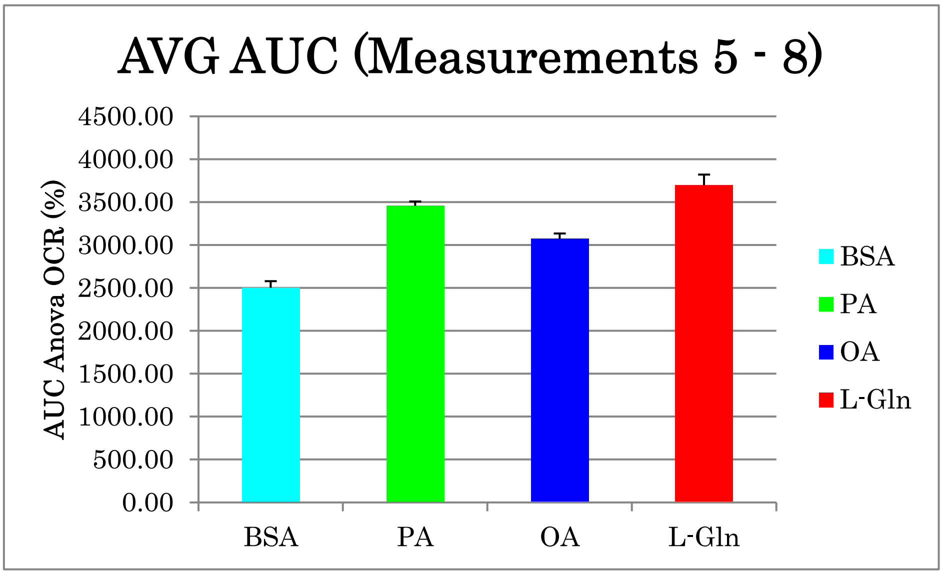

P Value (Tukey Post test)

	BSA	PA	OA	L-Gln
**BSA**		**0.000000**	**0.000000**	**0.000000**
**PA**			**0.000009**	**0.001646**
**OA**				**0.000000**

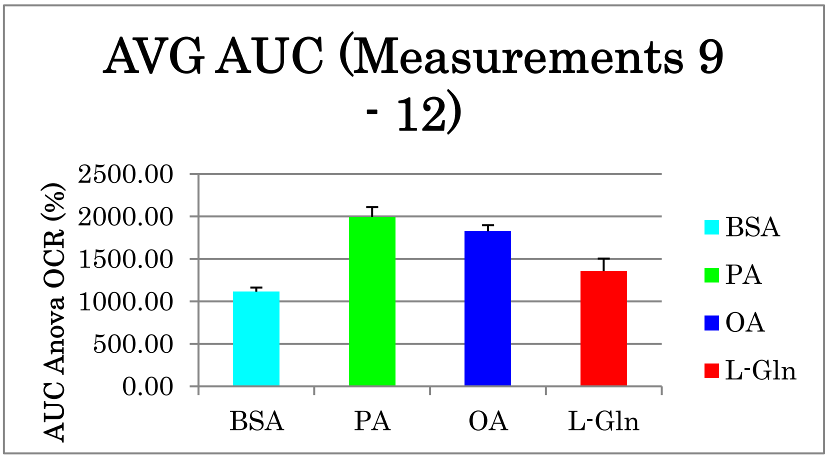

P Value

(Tukey Post test)

	BSA	PA	OA	L-Gln
BSA		0.000000	0.000000	0.008261
PA			0.084290	0.000000
OA				0.000011

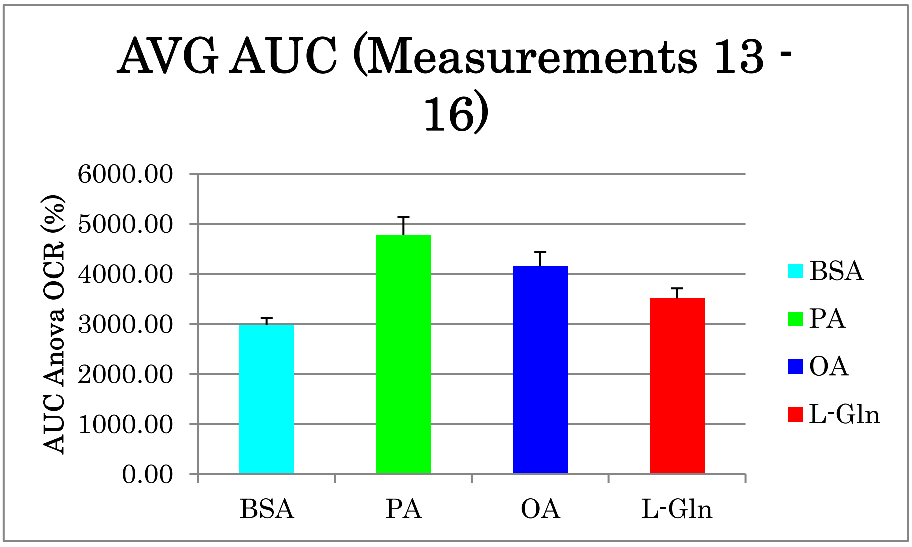

P Value

(Tukey Post test)

	**BSA**	**PA**	**OA**	**L-Gln**
**BSA**		**0.000000**	**0.000014**	**0.027260**
**PA**			**0.008460**	**0.000005**
**OA**				**0.006218**
