# Supplementary figures and images for: Rates of Mitochondrial Metabolism of Glucose, Amino Acids, and Fatty Acids by the HEI-OC1 Inner Ear Cell Line
Source: Biology (Basel). 2025 Aug 24;14(9):1118. doi: 10.3390/biology14091118 (PMC12467209; doi:10.3390/biology14091118)

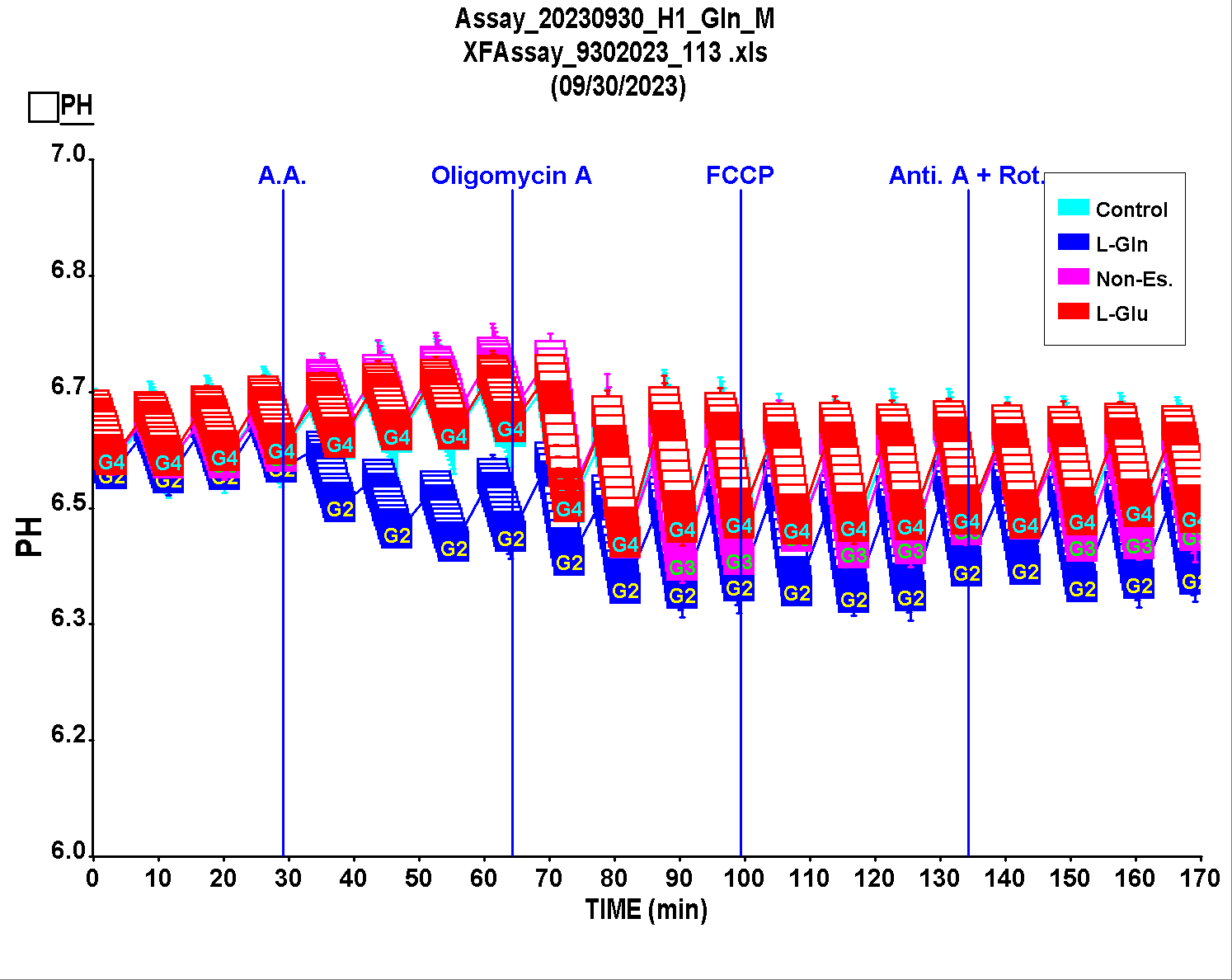

Supplement: Supplementary file 1 [file biology-14-01118-s001.zip › Suppl.S1 pH Raw Data/20230930 pH(related to Figure 3B).tif]

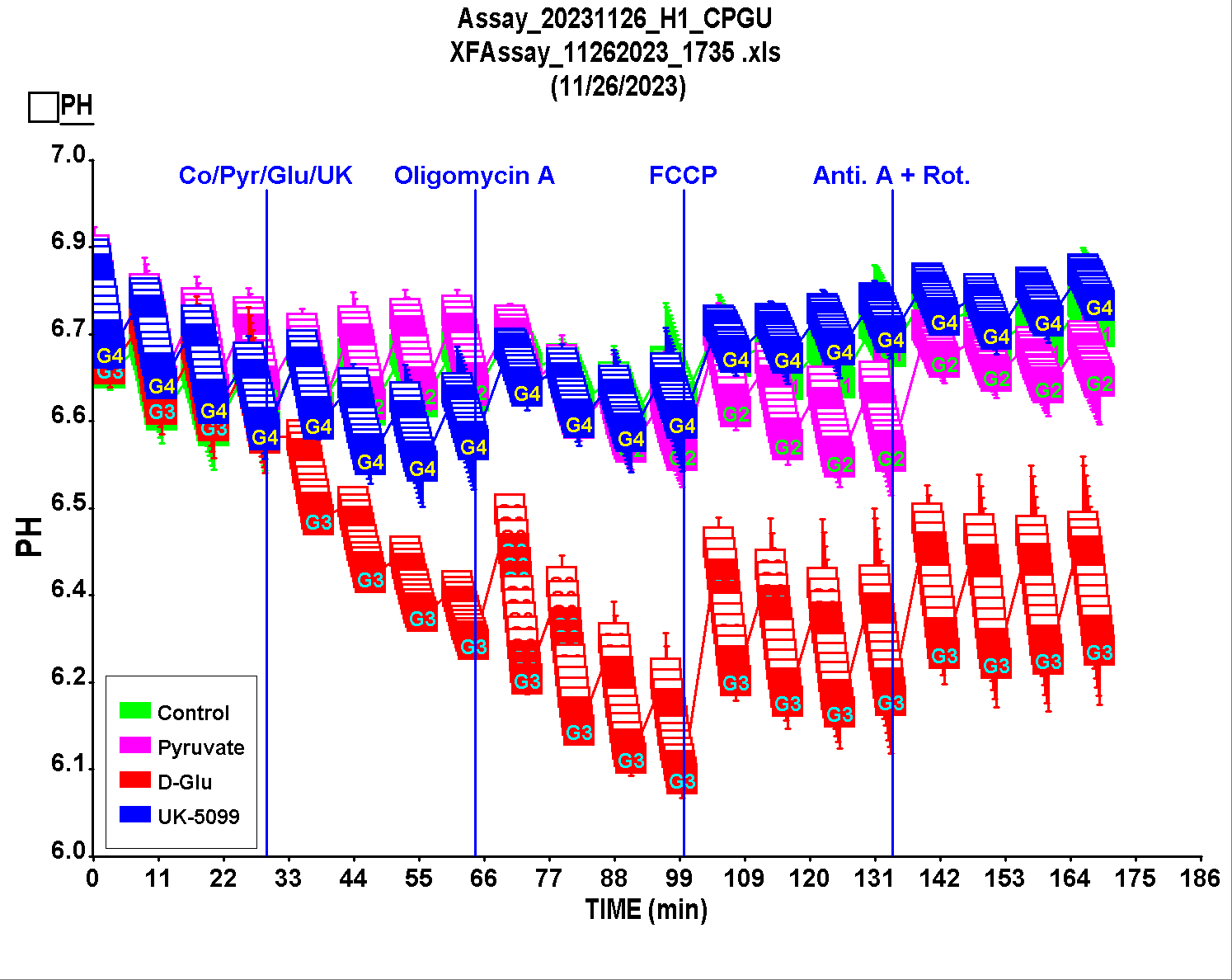

Supplement: Supplementary file 1 [file biology-14-01118-s001.zip › Suppl.S1 pH Raw Data/20231126 pH(related to Figure 2).tif]

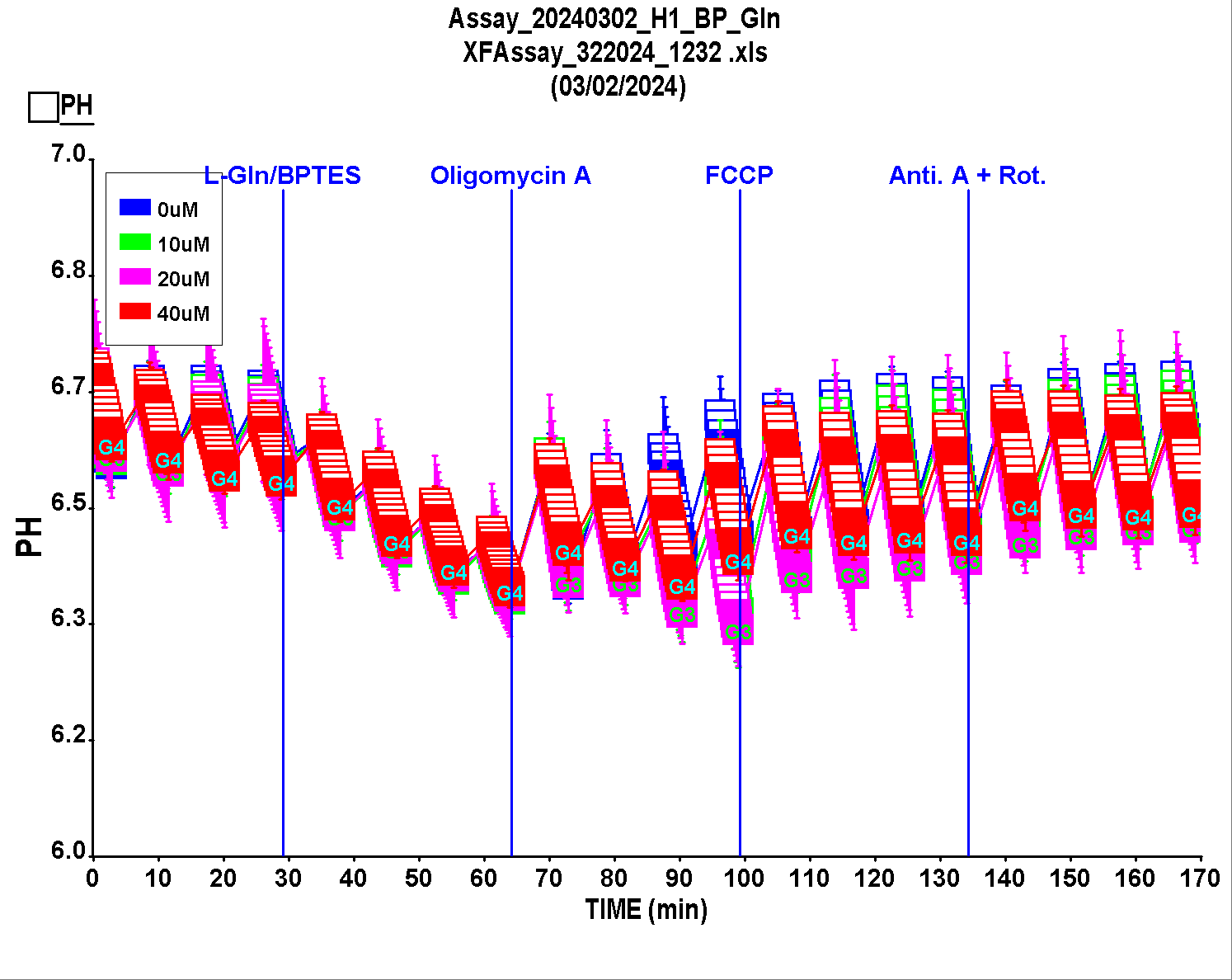

Supplement: Supplementary file 1 [file biology-14-01118-s001.zip › Suppl.S1 pH Raw Data/2024 0302 pH(related to Figure 4B).tif]

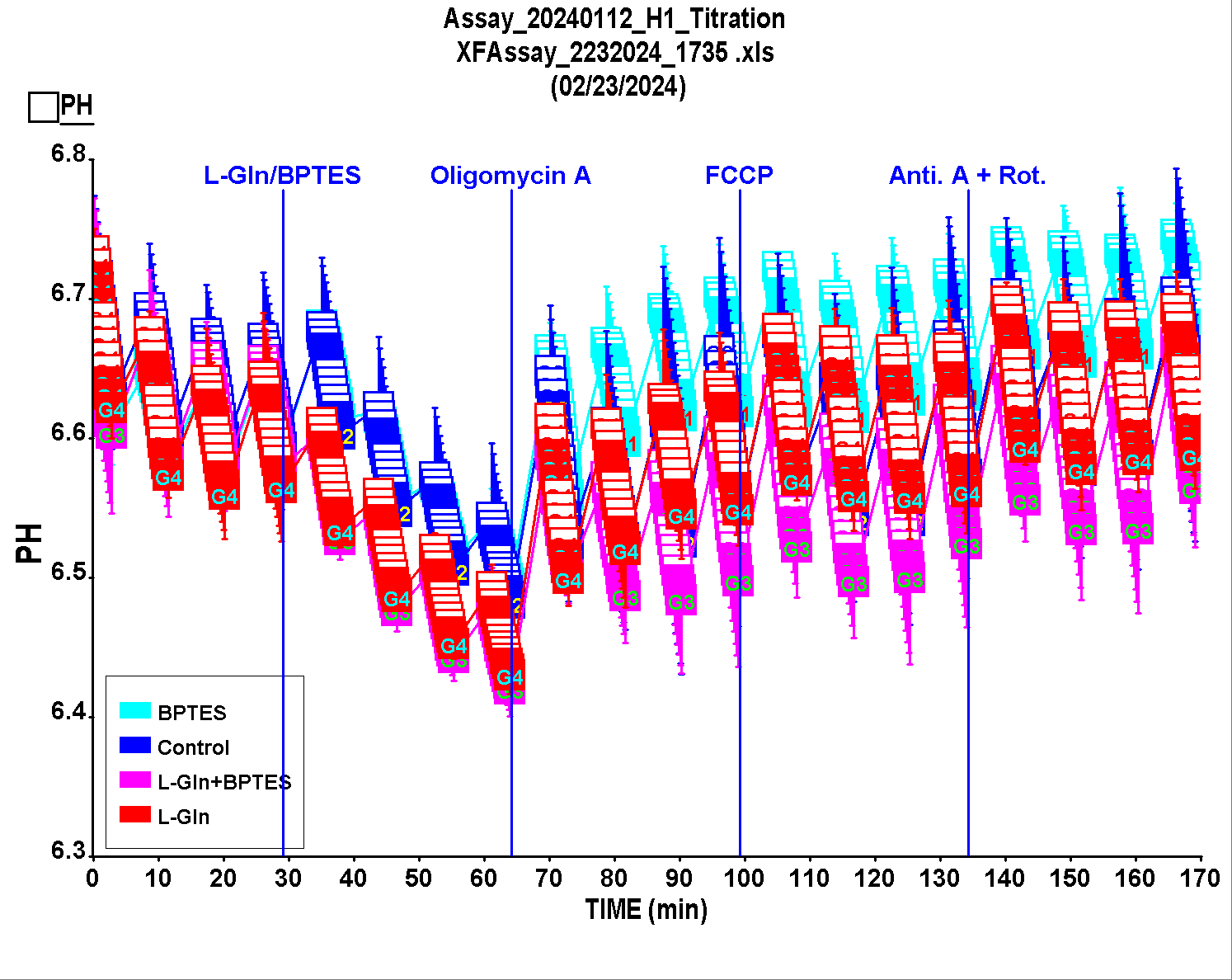

Supplement: Supplementary file 1 [file biology-14-01118-s001.zip › Suppl.S1 pH Raw Data/20240223 pH(related to Figure 4A).png]

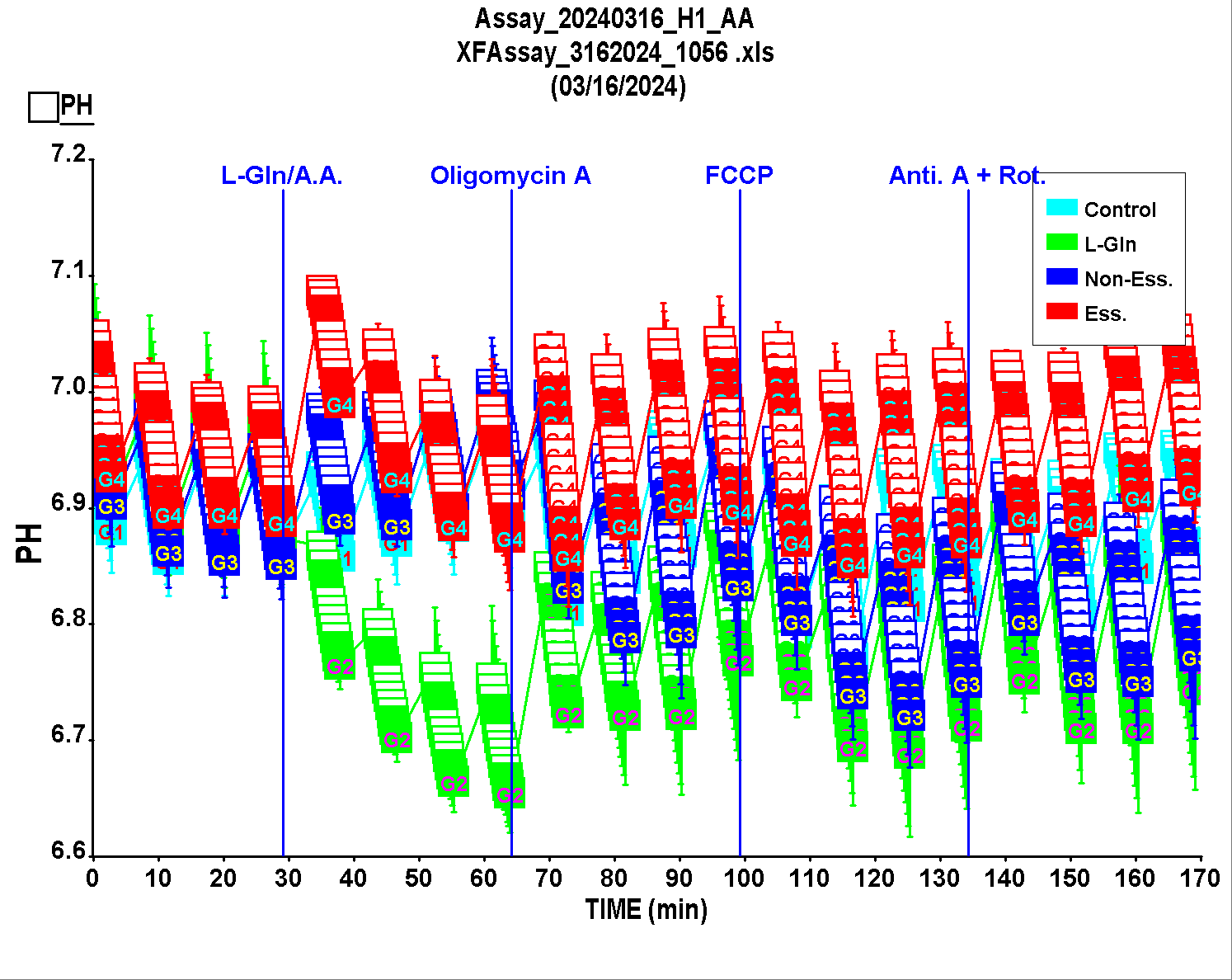

Supplement: Supplementary file 1 [file biology-14-01118-s001.zip › Suppl.S1 pH Raw Data/20240316pH(related to Figure 3A).png]

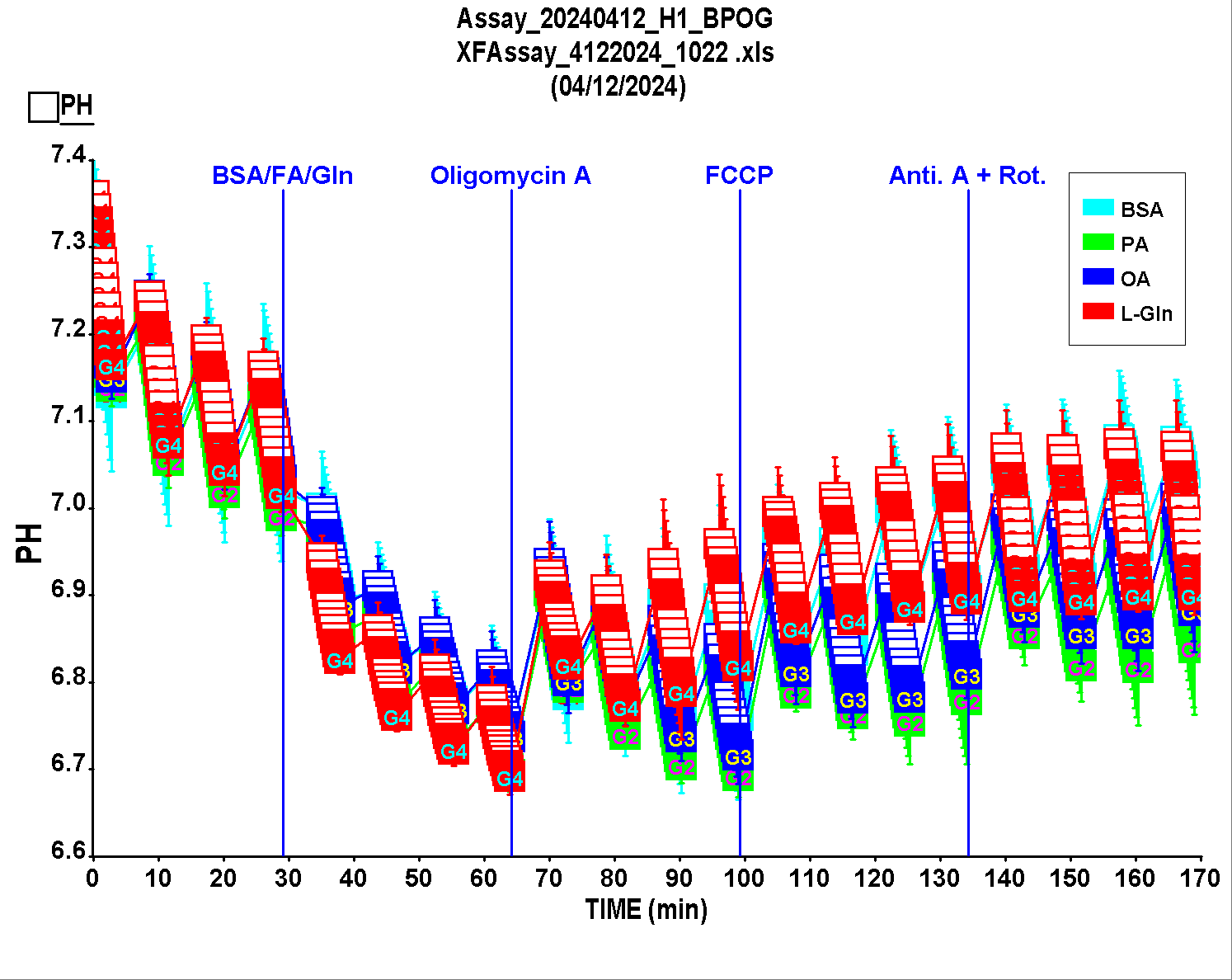

Supplement: Supplementary file 1 [file biology-14-01118-s001.zip › Suppl.S1 pH Raw Data/20240412pH(related to Figure 8).png]

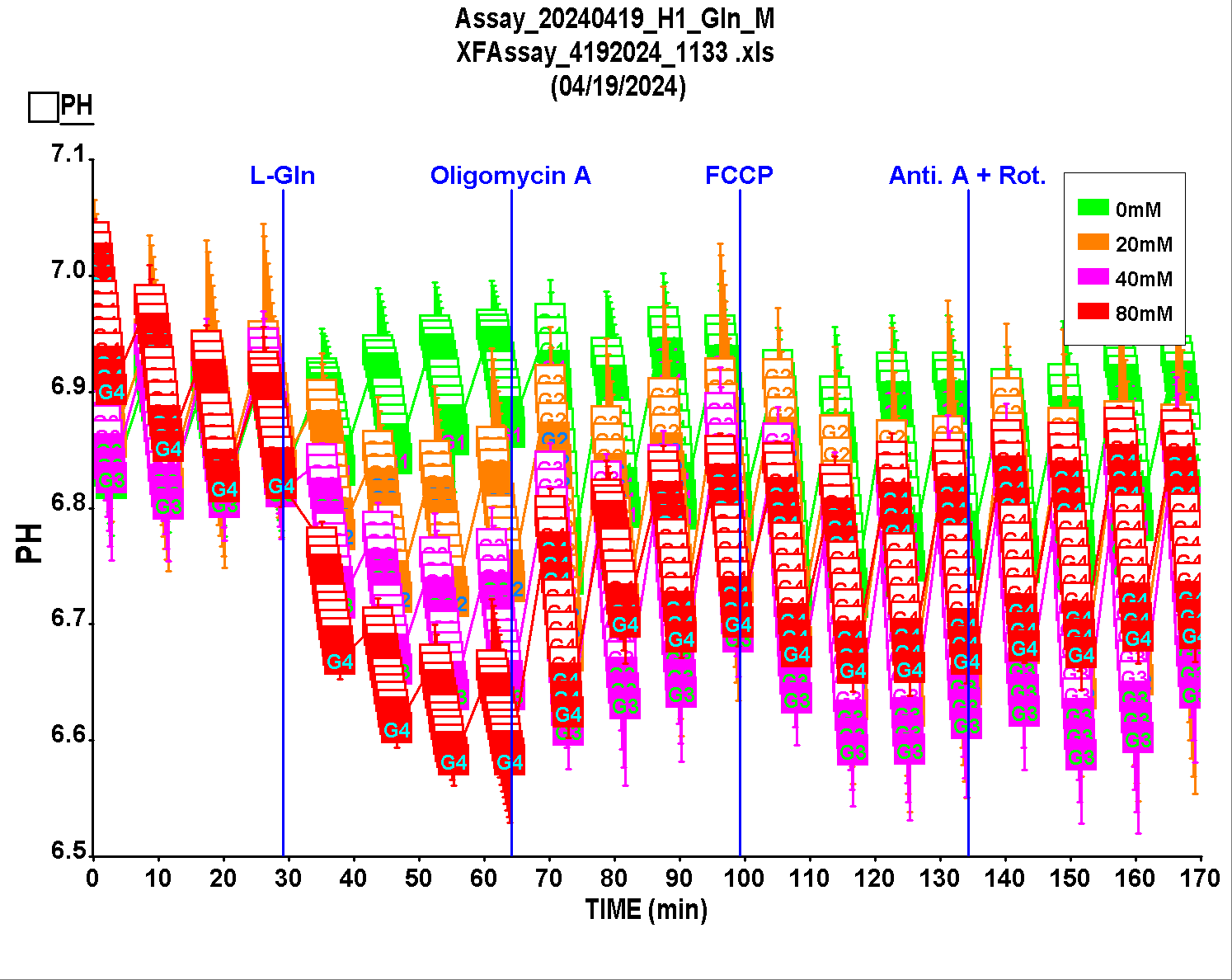

Supplement: Supplementary file 1 [file biology-14-01118-s001.zip › Suppl.S1 pH Raw Data/20240419pH(related to Figure 5A).png]

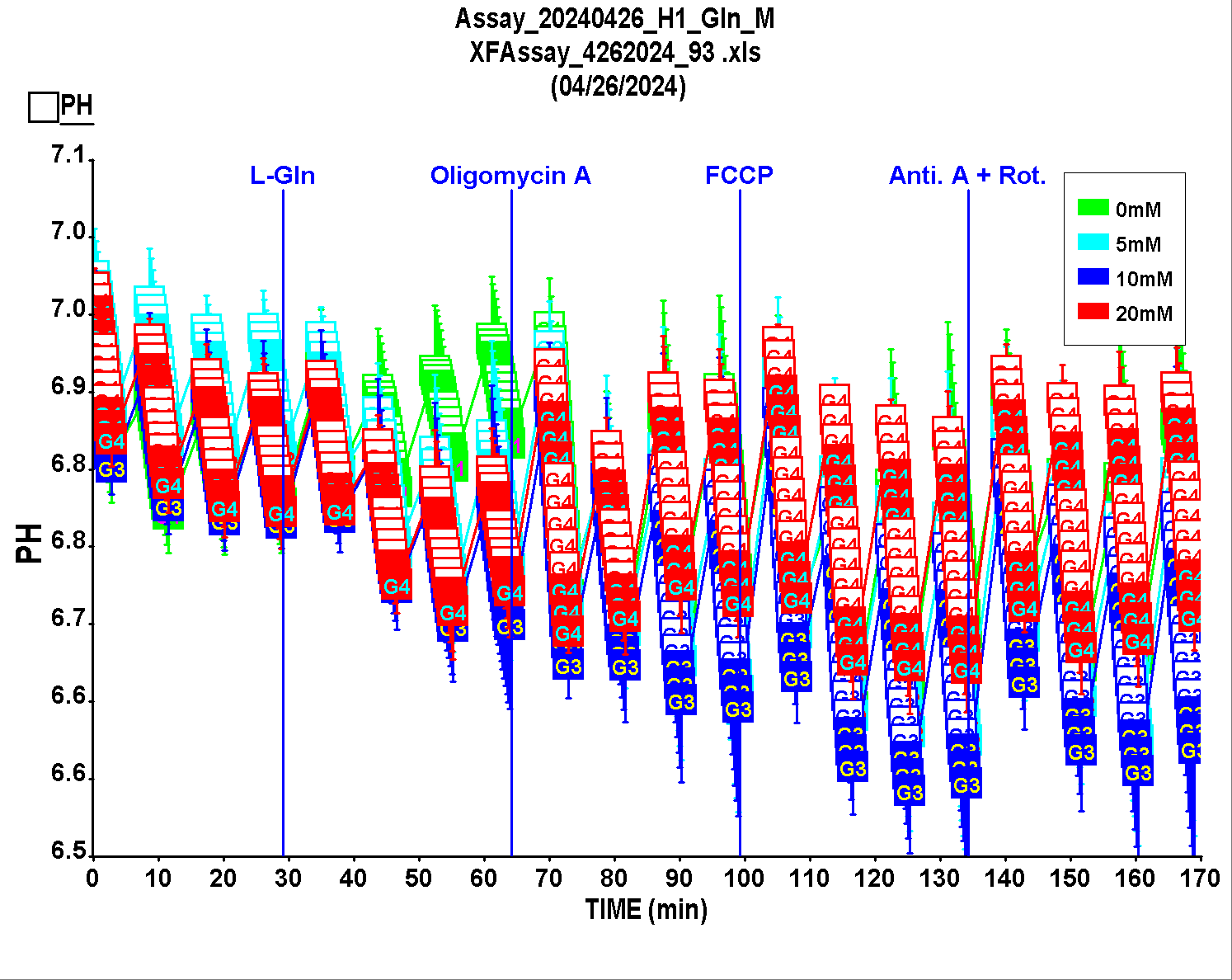

Supplement: Supplementary file 1 [file biology-14-01118-s001.zip › Suppl.S1 pH Raw Data/20240426pH(related to Figure 5B).png]

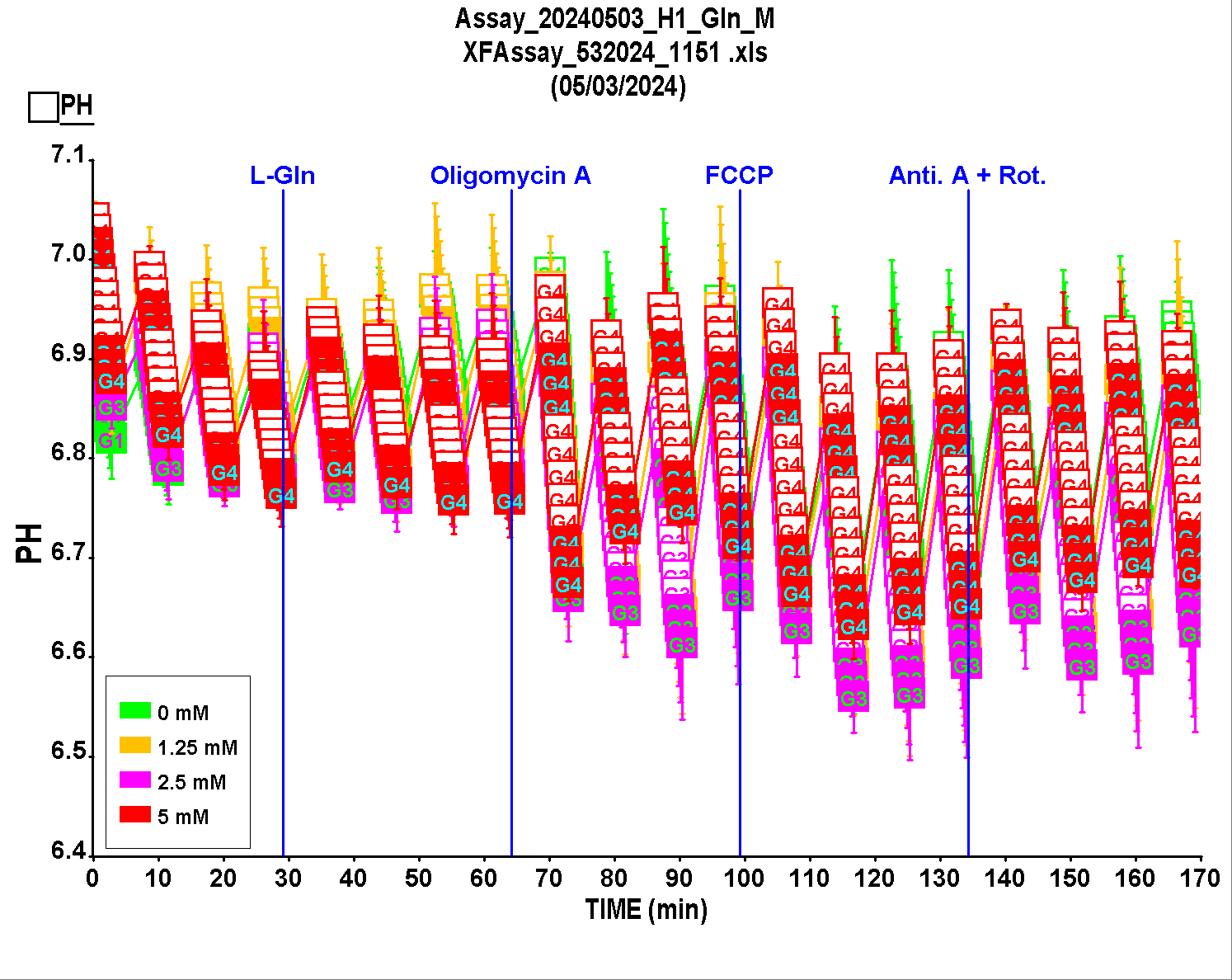

Supplement: Supplementary file 1 [file biology-14-01118-s001.zip › Suppl.S1 pH Raw Data/20240503 pH(related to Figure 5C).png]

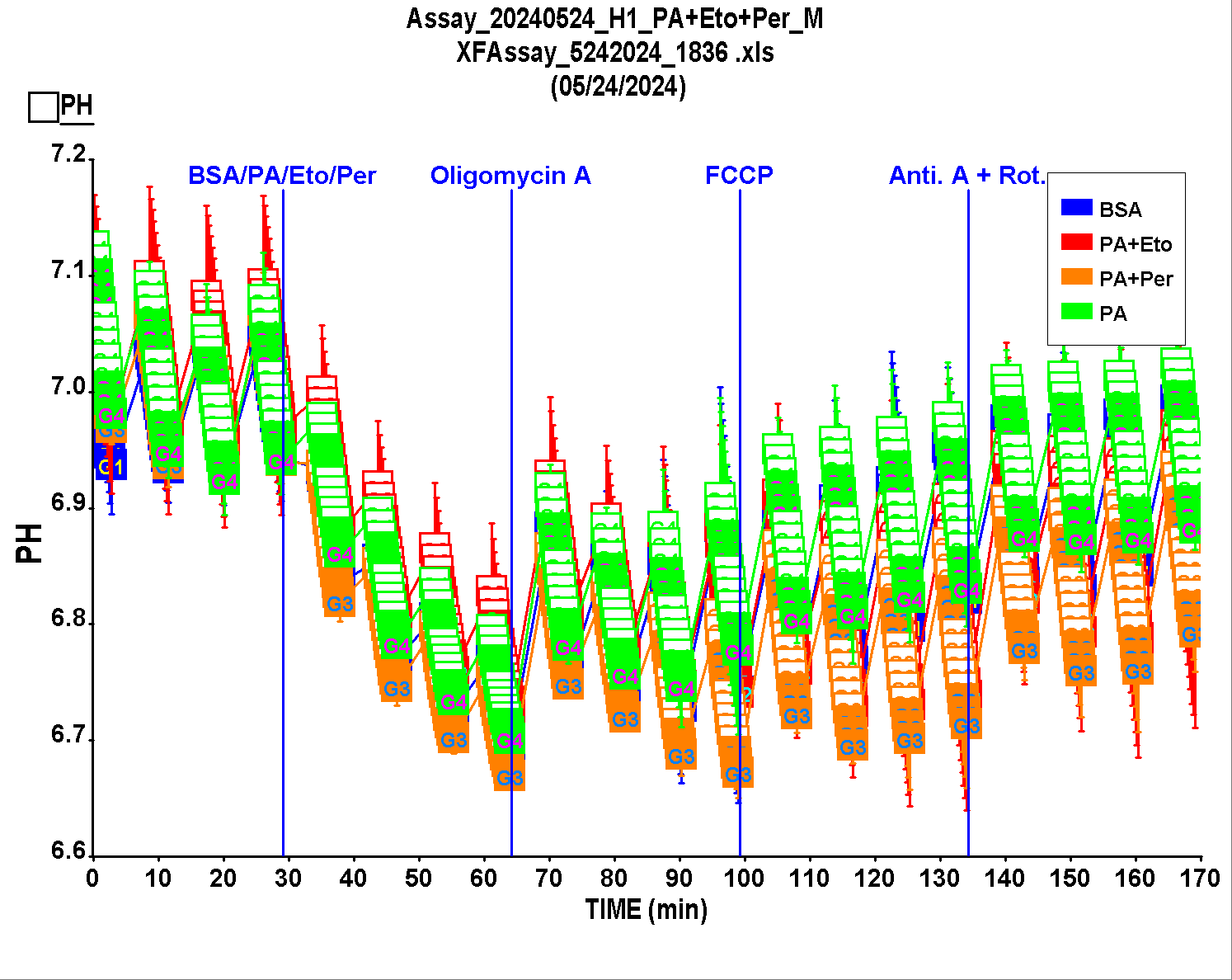

Supplement: Supplementary file 1 [file biology-14-01118-s001.zip › Suppl.S1 pH Raw Data/20240524pH(related to Figure 10B).png]

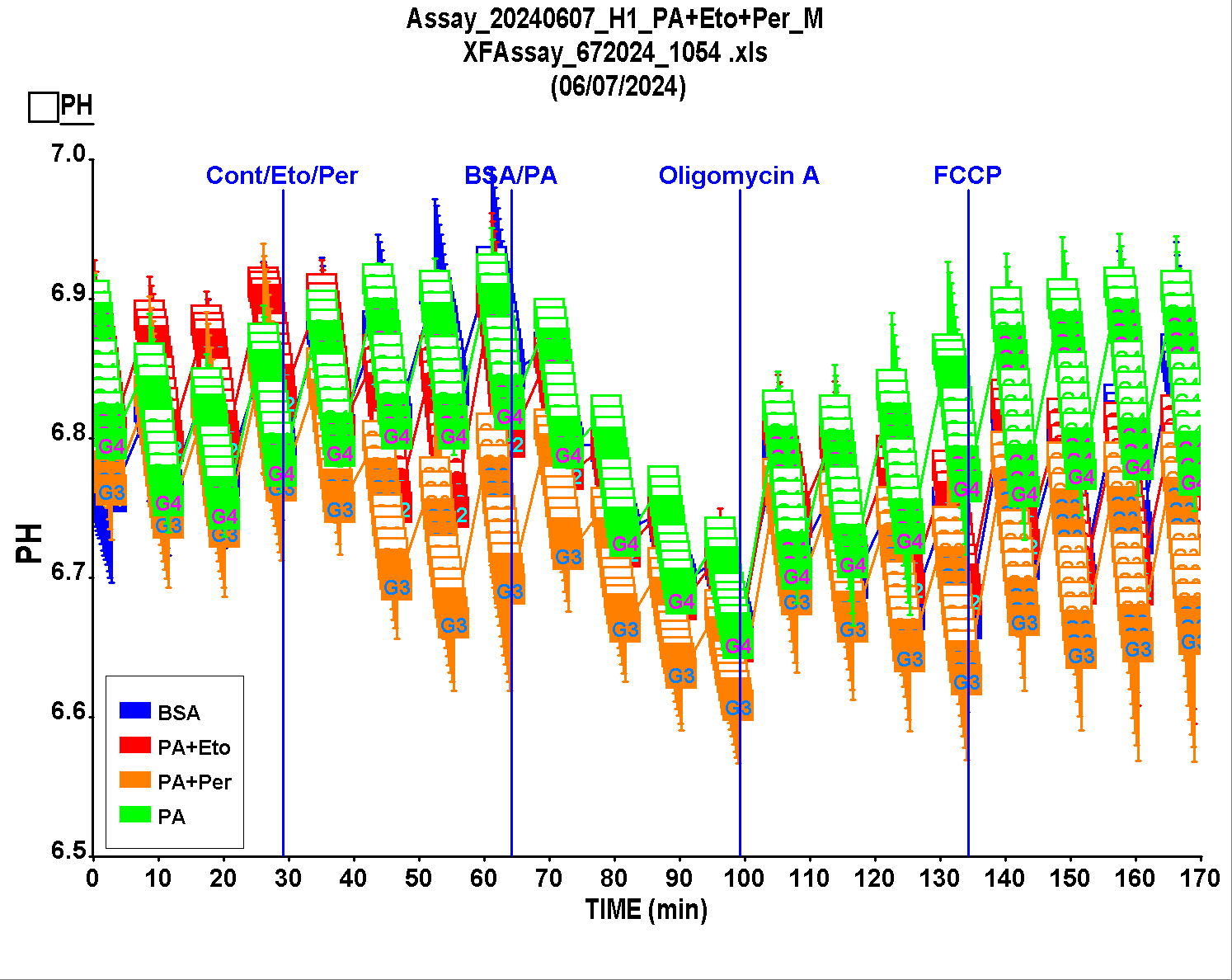

Supplement: Supplementary file 1 [file biology-14-01118-s001.zip › Suppl.S1 pH Raw Data/20240607pH(related to Figure 10A).png]
